# Engineering Pluripotent Stem Cells‐Derived Inner Ear Organoids With Enhanced Maturation and Reproducibility by Micro‐Topographical Cues

**DOI:** 10.1002/adma.74560

**Published:** 2026-08-10

**Authors:** Harshita Sharma, Jungeun Lim, Woochan Kim, Yeon Ju Kim, Dream Kim, Shinyull Lee, Chaeyeon Park, Stephen Rhee, Shruthy Kuttapan, Jungho Ha, Sang Wook Park, Kyunghoon Kim, Noo Li Jeon, Sunho Park, Yun‐Hoon Choung, Jangho Kim

**Affiliations:** ^1^ Department of Convergence Biosystems Engineering Chonnam National University Gwangju Republic of Korea; ^2^ Department of Rural and Biosystems Engineering Chonnam National University Gwangju Republic of Korea; ^3^ Interdisciplinary Program in IT‐Bio Convergence System Chonnam National University Gwangju Republic of Korea; ^4^ Department of Mechanical Engineering Seoul National University Seoul Republic of Korea; ^5^ Department of Neurosciences University of California San Diego CA USA; ^6^ Department of Neurology Graduate School of Medical Science Brain Korea 21 Project Yonsei University College of Medicine Seoul Republic of Korea; ^7^ Department of Otolaryngology Ajou University School of Medicine Suwon Republic of Korea; ^8^ Department of Oral Biochemistry School of Dentistry Chonnam National University Gwangju Republic of Korea; ^9^ School of Mechanical Engineering Sungkyunkwan University Suwon Republic of Korea; ^10^ Department of Bio‐Industrial Machinery Engineering Pusan National University Miryang‐si Republic of Korea; ^11^ Institute of Nano‐Stem Cells Therapeutics, NANOBIOSYSTEM Co., Ltd Gwangju Republic of Korea

**Keywords:** inner ear organoids, micro‐topographical cues, organoid reproducibility, pluripotent stem cells, sensory maturation, vascularized organoids

## Abstract

Inner ear organoids (IEOs) derived from pluripotent stem cells (PSCs) provide a promising platform for modeling neurosensory disorders and hearing loss; however, conventional systems often exhibit substantial structural variability, incomplete maturation, and limited reproducibility due to insufficient control of early organoid morphogenesis. Here, we demonstrate that micro‐topographical cues applied during initial IEO formation enhance the development and functional maturation of PSC‐derived IEOs. This microengineering strategy introduces temporally defined microscale geometric confinement to regulate early cell‐cell and cell‐extracellular matrix (ECM) interactions, thereby promoting epithelial organization and developmental fidelity. Microengineered IEOs (M‐IEOs) exhibit improved reproducibility and neurosensory maturation, including increased hair cell‐like populations, stereocilia‐like structures and kinocilium‐like features exhibiting a characteristic (9 × 2) + 2 microtubule organization. Functionally, M‐IEOs exhibit enhanced electrophysiological responsiveness, supported by complementary transcriptomic and in situ analyses indicating activation of inner ear lineage maturation pathways. Furthermore, we demonstrate the versatility of M‐IEOs by integrating them with a microfluidic vascular system to model vascular‐epithelial interactions and inflammatory responses, highlighting its potential for disease modeling and pharmacological screening. Together, these findings establish transient micro‐topographical guidance as an instructive regulator of inner ear organoid development and provide a robust, vascular‐compatible platform for neurosensory research, disease modeling, and translational screening applications.

## Graphical abstract

Micro‐topographical cues applied through temporally controlled microscale confinement improve the reproducibility, spatial organization, and neurosensory‐associated features of pluripotent stem cell‐derived inner ear organoids. Integration with a vascularized organoid platform further enables controlled investigation of vascular‐epithelial interactions and inflammatory responses in vitro.

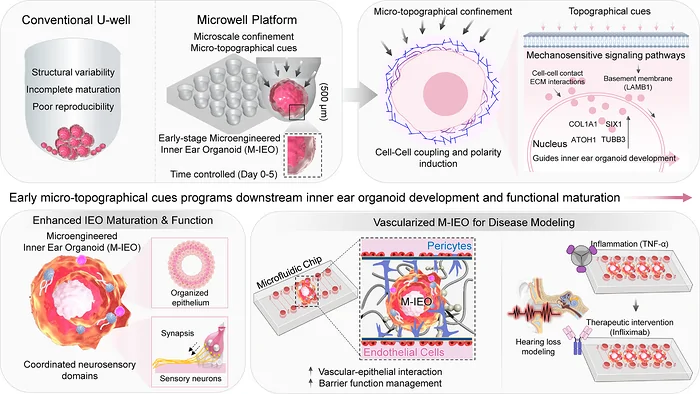

## 1 Introduction

Pluripotent stem cell‐derived inner ear organoids (IEOs) provide in vitro models to study key aspects of inner ear development, including the generation of hair cell‐like populations, supporting cells, and neurosensory features [1, 2, 3]. These systems enable investigation of otic lineage specification and sensory epithelial formation [4]. However, current IEO models do not fully recapitulate the structural organization and functional maturation of the native inner ear [5]. Organoids often exhibit variability in early aggregation, incomplete epithelial patterning, and immature sensory phenotypes, which limit their reproducibility and physiological relevance [6]. One contributing factor underlying this variability is insufficient control over early spatial and physical cues during organoid morphogenesis [7, 8]. Variations in cell aggregation, spatial organization, and local signaling environments can propagate through later stages of development, leading to heterogeneous tissue architectures and inconsistent functional outcomes [9]. Despite significant advances in organoid technologies, systematic regulation of these early developmental events remains a critical challenge in engineering complex tissues in vitro [6, 10].

During early development, spatial organization and topographical cues regulate cell‐cell and cell‐extracellular matrix (ECM) interactions that coordinate tissue patterning and lineage specification [11, 12, 13]. Micro‐topographical cues are defined as microscale physical and geometrical features that regulate cellular organization and behavior, encompassing a broad range of architectures, including grooves, ridges, pillars, pits, and confined microwells [14]. Previous studies have demonstrated that engineered micro‐ and nano‐topographical features regulate cellular organization, adhesion, cytoskeletal remodeling, and differentiation by modulating biomechanical and spatial cues within the cellular microenvironment [15, 16, 17]. Consistent with these principles, recent advances in three‐dimensional organoid engineering have further highlighted the importance of early spatial organization in directing lumen formation, tissue architecture, and morphogenesis [18, 19]. Therefore, engineering defined micro‐topographical cues provides an effective strategy for regulating cell fate and tissue morphogenesis. Previous studies have primarily utilized microwell‐based platforms as a culture tool to generate uniform embryoid bodies, spheroids, and initial organoid aggregates [20, 21, 22, 23, 24]. However, there has been limited investigation into how the micro‐topographical cues provided by these platforms during early morphogenesis influence subsequent organoid development, tissue organization, and functional maturation.

Building upon these principles, we present a transient micro‐topographical engineering strategy that applies temporally controlled microscale geometric confinement during the early stage of organoid aggregation to systematically investigate how early micro‐topographical cues regulate subsequent inner ear organoid development. We demonstrate that transient micro‐topographical regulation durably enhances organoid reproducibility, cell‐ECM organization, sensory epithelial architecture, mechanotransduction‐associated remodeling, and functional maturation, resulting in organized sensory epithelia with increased hair cell‐like populations, stereocilia‐like structures, enhanced electrophysiological activity, and coordinated activation of sensory and neuronal lineage programs. Furthermore, we extend this strategy by integrating microengineered inner ear organoids with a microfluidic vascular system to establish a vascularized inner ear model capable of recapitulating vascular‐epithelial interactions and inflammatory responses. Collectively, these findings establish transient micro‐topographical regulation as an instructive engineering strategy for directing PSC‐derived inner ear organoid development and provide a robust vascular‐compatible platform for disease modeling, mechanistic studies, and therapeutic screening.

## 2 Results

### 2.1 Transient Micro‐Topographical Confinement Improves Early PSC Aggregation, Uniformity, and Cell Viability

To investigate the influence of transient micro‐topographical regulation during the earliest stage of inner ear organoid development, we established a microengineered strategy that applies temporally controlled microscale geometric confinement during the initial aggregation phase (Day 0–5), followed by conventional suspension culture for subsequent differentiation (Figure 1a) [25]. The platform was fabricated using a soft lithography‐based replica molding process to generate arrays of uniform hemispherical PDMS microwells that provide controlled micro‐topographical cues during the initial stages of organoid formation (Figure 1b,c). Structural characterization by optical imaging and scanning electron microscopy (SEM) confirmed the uniform geometry and microscale architecture of the fabricated platform (Figure 1d). Unlike conventional U‐well systems, in which aggregates form through passive sedimentation, the microengineered platform provides defined microscale geometric confinement during the initial aggregation stage, enabling controlled cell organization and reproducible aggregate formation before subsequent suspension culture [26].

**FIGURE 1:**
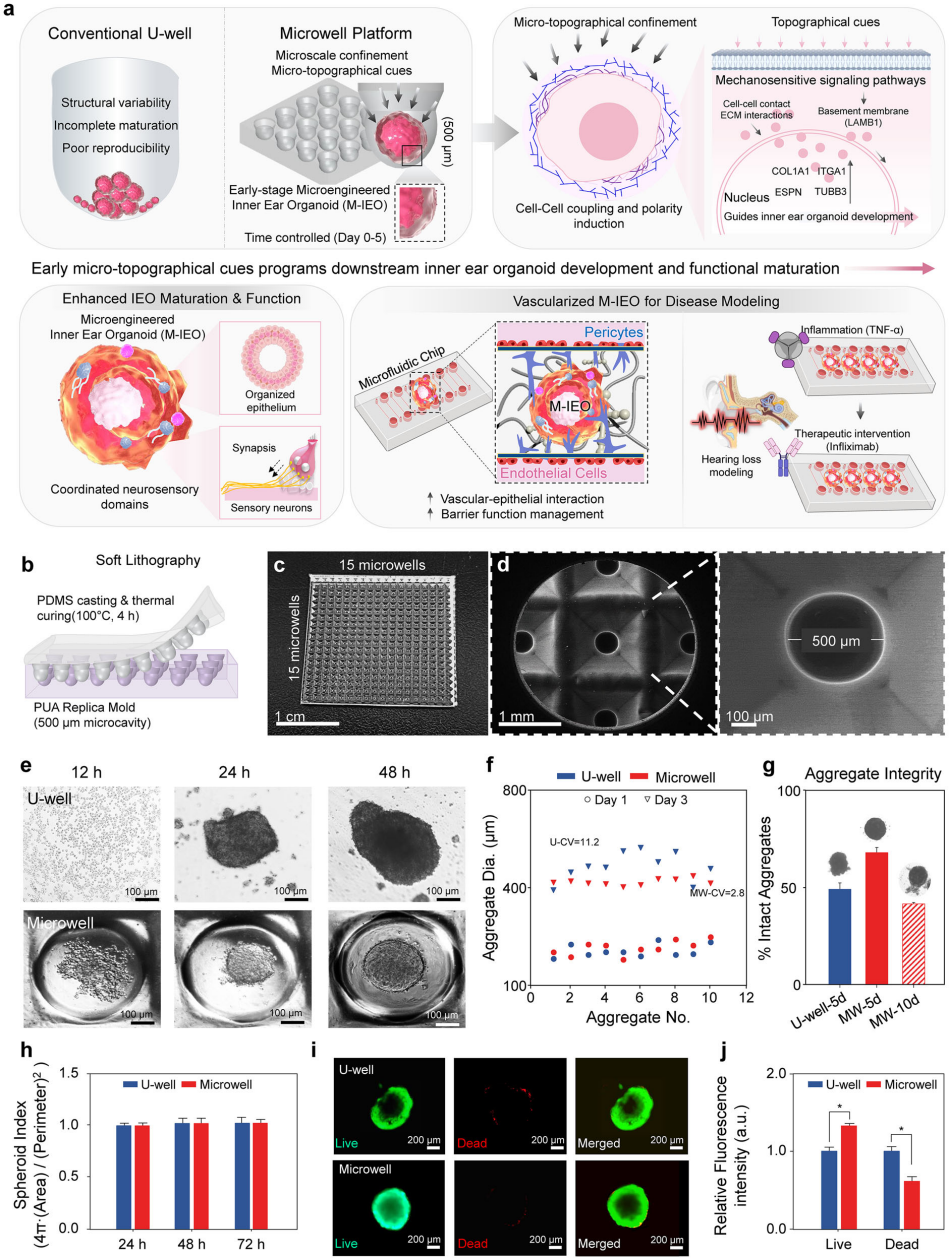
Temporally controlled micro‐topographical aggregation enables uniform PSC spheroid formation. a) Schematic illustration of the transient micro‐topographical engineering strategy. Compared with conventional U‐bottom culture, the microengineered platform provides defined microscale geometric confinement during the initial aggregation phase (Day 0–5) under identical induction conditions (cell seeding density, differentiation medium, morphogen treatment, and culture handling), without centrifugation. Early micro‐topographical regulation promotes controlled organoid assembly, resulting in enhanced structural organization, functional maturation, and subsequent integration with a vascular co‐culture platform. b) Schematic of the soft lithography‐based fabrication process used to generate the microengineered PDMS microwell platform through PUA replica molding. c) Representative photograph of the fabricated PDMS microwell platform, demonstrating the uniform hemispherical microwell architecture (500 µm diameter) used to provide defined micro‐topographical cues during early organoid aggregation. d) Macroscopic and SEM images of the microwell array (left, center) and a single 500 µm microwell (right). e) Time‐course bright‐field images showing aggregation of mouse embryonic stem cells (mESCs) within microwells from 12 h to 48 h. Scale bar, 100 µm. f) Aggregate diameter distribution of Day 3 aggregates generated in U‐well and microwell cultures (*n* = 10 aggregates per group). g) Quantification of aggregate integrity following different durations of microwell confinement, demonstrating the highest percentage of intact aggregates at 5 days compared to U‐well controls and prolonged confinement (10 days). h) Spheroid index at 24, 48, and 72 h. i) Representative Live/Dead staining images of aggregates from U‐well and microwell cultures. Scale bar, 200 µm. j) Quantification of Live (green) and Dead (red) fluorescence intensity at 72 h (*n* = 3 independent biological replicates). Data are presented as mean ± s.e.m. from n ≥ 3 independent biological replicates. Statistical tests are indicated in each panel. *p < 0.05, **p < 0.01, ***p < 0.001.

To identify optimal aggregation conditions, we evaluated the effect of initial seeding density (2 × 10^3^, 3 × 10^3^, and 4 × 10^3^ cells per well; MS‐, MM‐, and ML‐Sph, respectively) without centrifugation to ensure consistent handling. While all conditions formed aggregates within 24 h, U‐well cultures frequently generated multiple or irregular clusters per well. In contrast, microwell‐based aggregation produced single, spatially confined spheroids with uniform size and distribution. The highest density condition (ML‐Sph) yielded more compact and structurally stable aggregates with reduced variability over time (Figure S1a–e). Immunofluorescence analysis revealed enhanced cytoskeletal organization and epithelial characteristics in ML‐Sph aggregates, indicated by increased phalloidin and integrin β4 (ITGB4) expression (Figure S1f,g). Following Matrigel supplementation, these aggregates developed a dense peripheral layer with basement membrane‐like features, supported by SEM observations and increased laminin β1 (LAMB1) expression alongside reduced OCT4 levels (Figure S2a–f). Additionally, connexin 43 (CX43) expression increased over time, while Cyclin D1 (CCND1) decreased, consistent with progression toward a more differentiated state (Figure S2g,h).

To determine whether differences established during early aggregation influenced subsequent organoid development, aggregates generated under the three seeding densities were cultured to Day 15 (Figure S3a,b). Whereas the MS‐Sph and MM‐Sph groups exhibited limited epithelial organization, ML‐Sph aggregates developed well‐defined otic‐like epithelial structures with increased expression of the otic marker PAX8 compared with the lower‐density groups and conventional U‐well controls (Figure S3c–f). Based on these findings, the ML‐Sph condition was selected as the optimized aggregation strategy for all subsequent experiments.

Using the optimized ML‐Sph condition, aggregation outcomes were directly compared between microwell and U‐well formats. Under identical conditions, U‐well cultures showed heterogeneous aggregation, while microwell cultures consistently produced single, centrally confined spheroids (Figure 1e). Quantitative analysis demonstrated that microwell‐derived aggregates exhibited a markedly lower coefficient of variation than U‐well aggregates (CV: 2.8% vs. 11.2%), indicating significantly improved size uniformity during the initial stage of organoid formation (Figure 1f). To investigate the temporal role of topographical confinement, aggregates were maintained within microwells for defined durations prior to release (Figure 1g). Short‐term confinement (5 days) resulted in a higher proportion of intact, cohesive aggregates compared to U‐well controls. In contrast, prolonged confinement (10 days) led to a reduction in aggregate integrity following release. These results indicate that the duration of early spatial confinement influences subsequent structural outcomes and suggest the existence of a temporal window during which topographical regulation is most effective for maintaining aggregate integrity. Consistently, microwell‐derived spheroids exhibited increased compactness compared to U‐well aggregates, while both conditions reached comparable circularity by 72 h (Figure 1h). Live/Dead staining further confirmed improved viability, with increased live‐cell signal and reduced cell death in microwell‐derived aggregates (Figure 1i,j). Collectively, these results demonstrate that temporally defined micro‐topographical confinement during early aggregation reduces variability and establishes a critical window that governs spheroid stability and integrity. Following this transient aggregation period, all spheroids were transferred to identical suspension culture conditions and subjected to the same differentiation protocol, allowing the long‐term developmental effects of early micro‐topographical confinement to be evaluated independently of subsequent culture conditions. This optimized strategy was subsequently applied to generate microengineered inner ear organoids (M‐IEOs) and compared with U‐well‐derived inner ear organoids (U‐IEOs), with the initial aggregation format representing the only experimental variable.

### 2.2 Early Micro‐Topographical Control Enhances Otic Differentiation and Sensory Features in M‐IEOs

To investigate whether early micro‐topographical confinement influences downstream otic differentiation, PSC aggregates were subjected to a stepwise directed differentiation protocol toward inner ear organoids (Figure 2a) [27]. This induction strategy guided mouse embryonic stem cells (mESCs) through sequential stages from ectodermal specification to sensory epithelial development. During early differentiation (Day 1–3), organoids formed under microwell confinement (M‐mIEOs) maintained a consistent spherical morphology following Matrigel supplementation, whereas U‐well‐derived organoids (U‐mIEOs) frequently exhibited irregular shapes and partial fragmentation (Figure 2b). By Day 5, immunofluorescence analysis indicated progression toward a non‐neural ectodermal state in both conditions, as reflected by reduced OCT4 expression and increased Laminin β1 signals (Figures 2c,d). While both groups exhibited comparable lineage transitions, marker distribution appeared more spatially uniform in M‐mIEOs. To further examine developmental dynamics, temporal RT‐qPCR analysis was performed across key differentiation stages. M‐mIEOs exhibited more consistent expression trajectories of stage‐associated genes, including *Lamb1* (basement membrane), *Gata2* (non‐neural ectoderm), *Six1* and *Pax8* (pre‐placodal/otic lineage), and *Pax2* and *Jag1* (otic vesicle/prosensory domain), compared to U‐mIEOs (Figure 2e, primer sequences provided in Table S1) [28]. These findings indicate reduced variability and more synchronized lineage progression under topographically confined conditions.

**FIGURE 2:**
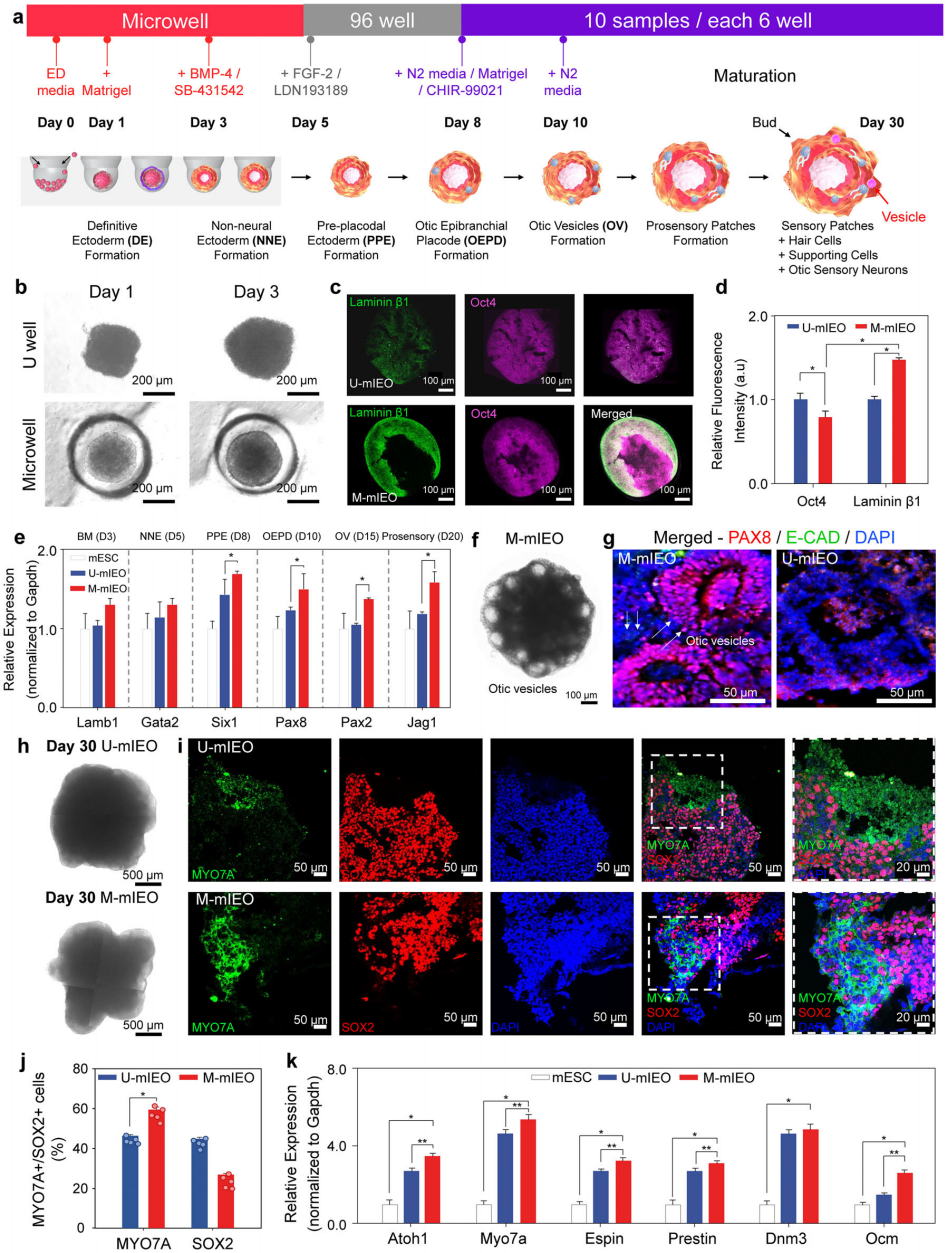
Early micro‐topographical control enables directed differentiation and synchronized otic lineage progression. a) Schematic of the stepwise induction protocol for mouse inner ear organoids (mIEOs). b) Bright‐field images of U‐mIEOs and M‐mIEOs at Day 1 and Day 3. Scale bars, 200 µm. c) Immunofluorescence staining for Laminin β1 (green) and OCT4 (magenta) at Day 5. d) Relative fluorescence intensity of Laminin β1 and OCT4 at Day 5. e) Temporal RT‐qPCR analysis of stage‐associated markers (*Lamb1*, *Gata2, Six1, Pax8, Pax2, Jag1*) from Day 5 to Day 20. f,g) Bright‐field and immunofluorescence images of epithelial vesicle‐like structures at Day 14, stained for PAX8 (red), E‐CAD (green), and DAPI (blue). Scale bars, 100, 50 µm. h) Bright‐field images of organoids at Day 30 showing complex morphologies. Scale bars, 500 µm. i) Immunofluorescence staining showing regions expressing MYO7A (green) and SOX2 (red) at Day 30. Insets show higher magnification views. Scale bars, 50 µm (overview), 20 µm (insets). j) Quantification of MYO7A^+^ and SOX2^+^ signal across fields of view (*n* = 5 independent regions per condition). k) RT‐qPCR analysis of sensory lineage‐associated genes (*Atoh1, Myo7a, Espin, Prestin, Dnm3, Ocm*) at Day 30. Data are presented as mean ± s.e.m. from *n* ≥ 3 independent biological replicates. Statistical tests are indicated in each panel. *p < 0.05, **p < 0.01, ***p < 0.001.

By Day 14, M‐mIEOs developed epithelial vesicle‐like structures characterized by PAX8 and E‐CAD expression with discernible luminal organization, whereas U‐mIEOs displayed less organized epithelial morphology (Figures 2f,g). Quantitative analysis further supported improved consistency in organoid size and vesicle formation in M‐mIEOs (Figure S4a–f). At later stages (Day 30), both conditions generated complex organoid structures with multiple epithelial protrusions (Figure 2h). Immunofluorescence analysis revealed regions expressing MYO7A and SOX2 in both groups (Figure 2i) [29, 30]. Consistent with known limitations in marker specificity and localization, these signals are interpreted as indicative of hair cell‐associated and supporting/progenitor‐like features rather than definitive cell‐type identity. Notably, M‐mIEOs exhibited a higher proportion of MYO7A^+^ regions compared to U‐mIEOs (Figure 2j), suggesting enrichment of sensory‐associated domains under microengineered conditions. To further characterize these populations, RT‐qPCR analysis was performed for a panel of sensory‐associated genes. M‐mIEOs showed increased expression of *Atoh1, Myo7a*, and *Espin*, along with additional markers such as *Prestin, Dnm3*, and *Ocm* (Figure 2k). Collectively, these results demonstrate that transient topographical confinement during early aggregation synchronizes otic lineage progression, reduces developmental variability, and promotes enrichment of sensory‐associated characteristics [31].

### 2.3 Micro‐Topographical Cues Enhances Ultrastructural Organization and Functional Properties of IEOs

To assess the emergence of neuronal‐ and sensory‐associated features, we first examined marker expression within organoids. Immunofluorescence staining for TUJ1 and MYO7A revealed more extensive TUJ1‐positive projections in M‐mIEOs compared to U‐mIEOs (Figure 3a,b). Consistently, RT‐qPCR analysis showed increased expression of neuronal‐associated genes, including *Nestin, Tubb3*, and *NeuN*, in M‐mIEOs (Figure 3c). In addition, elevated expression of synaptic‐associated genes (*Slc17a8, Syn1*) was observed (Figure 3d), suggesting enhanced neuronal differentiation and synaptic gene expression [32]. To evaluate membrane‐associated functional properties, FM1‐43 uptake assays were performed [33]. M‐mIEOs exhibited increased dye uptake compared to U‐mIEOs (Figure 3e,f), indicating the presence of cells with membrane permeability consistent with previously reported mechanosensitive uptake mechanisms in sensory epithelial systems [34]. High‐magnification imaging revealed F‐actin‐rich apical protrusions in regions exhibiting MYO7A signal (Figure 3g). To further examine ultrastructural organization, transmission electron microscopy (TEM) was performed. Cells within M‐mIEOs displayed apical protrusions and ciliary structures, including kinocilium‐like features with a (9x2) + 2 microtubule arrangement (Figures 3h–j), whereas U‐mIEOs exhibited less organized apical surfaces. Quantitative analysis indicated increased kinocilium length in M‐mIEOs, consistent with enhanced apical structural organization.

**FIGURE 3:**
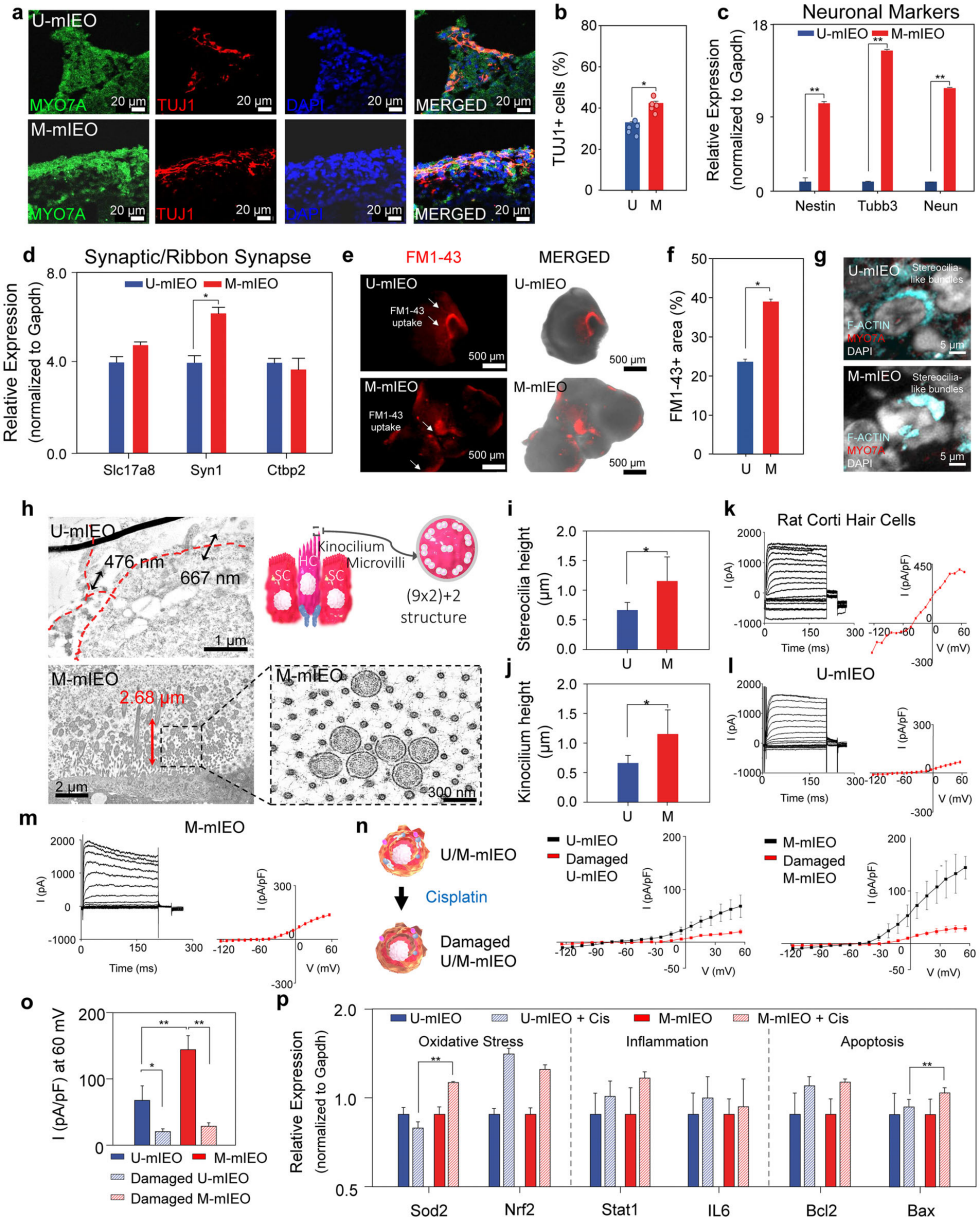
Ultrastructural, electrophysiological, and ototoxic stress response characterization of M‐mIEOs. a) Immunofluorescence staining showing regions expressing MYO7A (green) and TUJ1 (red). Scale bars, 20 µm. b) Quantification of TUJ1‐positive area (*n* = 5 independent regions per condition). (c and d) RT‐qPCR analysis of neuronal‐associated (c) and synaptic‐associated (d) genes, normalized to *Gapdh*. (e and f) Representative images (e) and quantification (f) of FM1‐43 uptake. Scale bars, 500 µm. g) High‐magnification immunofluorescence images showing F‐actin (cyan) and MYO7A (red) signal, highlighting apical protrusion‐like structures. Scale bars, 5 µm. h) Transmission electron microscopy (TEM) images showing apical structures in U‐mIEOs and M‐mIEOs. (i and j) Representative patch‐clamp traces and current–voltage (*I–V*) relationships for primary rat cochlear cells (k), U‐mIEO‐derived cells (l), and M‐mIEO‐derived cells (m). n) Schematic of cisplatin treatment and representative *I–V* curves following exposure. o) Quantification of peak current density before and after cisplatin treatment. p) RT‐qPCR analysis of stress‐ and apoptosis‐associated genes following treatment. Data are presented as mean ± s.e.m. from *n* ≥ 3 independent biological replicates. Statistical tests are indicated in each panel. *p < 0.05, **p < 0.01, ***p < 0.001.

To further evaluate whether these structural features translated into functional electrophysiological properties, whole‐cell patch‐clamp recordings were performed using dissociated organoid‐derived cells (Figure 3k–m). Cells were selected based on morphological characteristics under differential interference contrast (DIC) imaging; however, definitive cell identity could not be confirmed at the time of recording. Due to the limited yield of viable dissociated organoid‐derived cells exhibiting stable membrane properties suitable for recording, four cells (*n* = 4) were included for functional assessment. M‐mIEO‐derived cells exhibited voltage‐dependent outward currents, whereas U‐mIEO‐derived cells showed comparatively weaker responses (Figure 3l,m). Primary rat cochlear cells were included as a functional reference (Figure 3k). M‐mIEO‐derived cells showed a trend toward higher current density than U‐mIEO‐derived cells (Figure 3o). The recorded current amplitudes (20‐100 pA) were within the picoampere range, consistent with an early developmental electrophysiological profile previously reported for stem cell‐derived hair cell‐like cells [35].

To evaluate responses to ototoxic stress, organoids were treated with cisplatin [36]. Cisplatin treatment reduced current density in both groups. However, M‐mIEO‐derived cells retained higher current density than U‐mIEO‐derived cells following cisplatin exposure (Figure 3n,o), suggesting partial preservation of electrophysiological activity. This functional change was accompanied by increased expression of oxidative stress (*Sod2, Nrf2*), inflammatory (*Stat1, Il6*), and apoptosis‐associated (*Bcl2, Bax*) genes (Figure 3p). Immunofluorescence analysis further indicated a reduction in MYO7A‐associated signal following cisplatin exposure, while SOX2‐positive regions were relatively preserved (Figure S4g–h). Consistently, expression of sensory‐ and neuronal‐associated genes (*Espin, NeuN, Syn1, Slc17a8*) was reduced after treatment (Figure S4i). Collectively, these findings indicate that microengineered organoids exhibit enhanced development of sensory‐associated structural features together with emergent electrophysiological properties, while maintaining responsiveness to ototoxic perturbation.

### 2.4 Spatial Organization and Emergent Electrophysiological Activity in Human Inner Ear Organoids With Micro‐Topography

To assess the translational relevance of early micro‐topographical confinement, human induced pluripotent stem cells (hiPSCs) were differentiated into inner ear organoids (hIEOs) over a 90‐day protocol under microengineered (M‐hIEO) and conventional U‐well (U‐hIEO) conditions (Figure 4a,b) [37, 38]. Consistent with the findings in mouse organoids, M‐hIEOs exhibited improved structural integrity and reduced morphological variability throughout differentiation, indicating that transient geometric confinement during the initial aggregation stage exerts persistent effects on subsequent tissue organization. To investigate the structural organization of the resulting sensory epithelium, organoids were first examined by F‐actin (phalloidin) and DAPI staining. High‐resolution confocal imaging together with orthogonal and three‐dimensional reconstructions revealed that M‐hIEOs developed more continuous epithelial structures than U‐hIEOs, which displayed comparatively heterogeneous tissue organization (Figure 4c).

**FIGURE 4:**
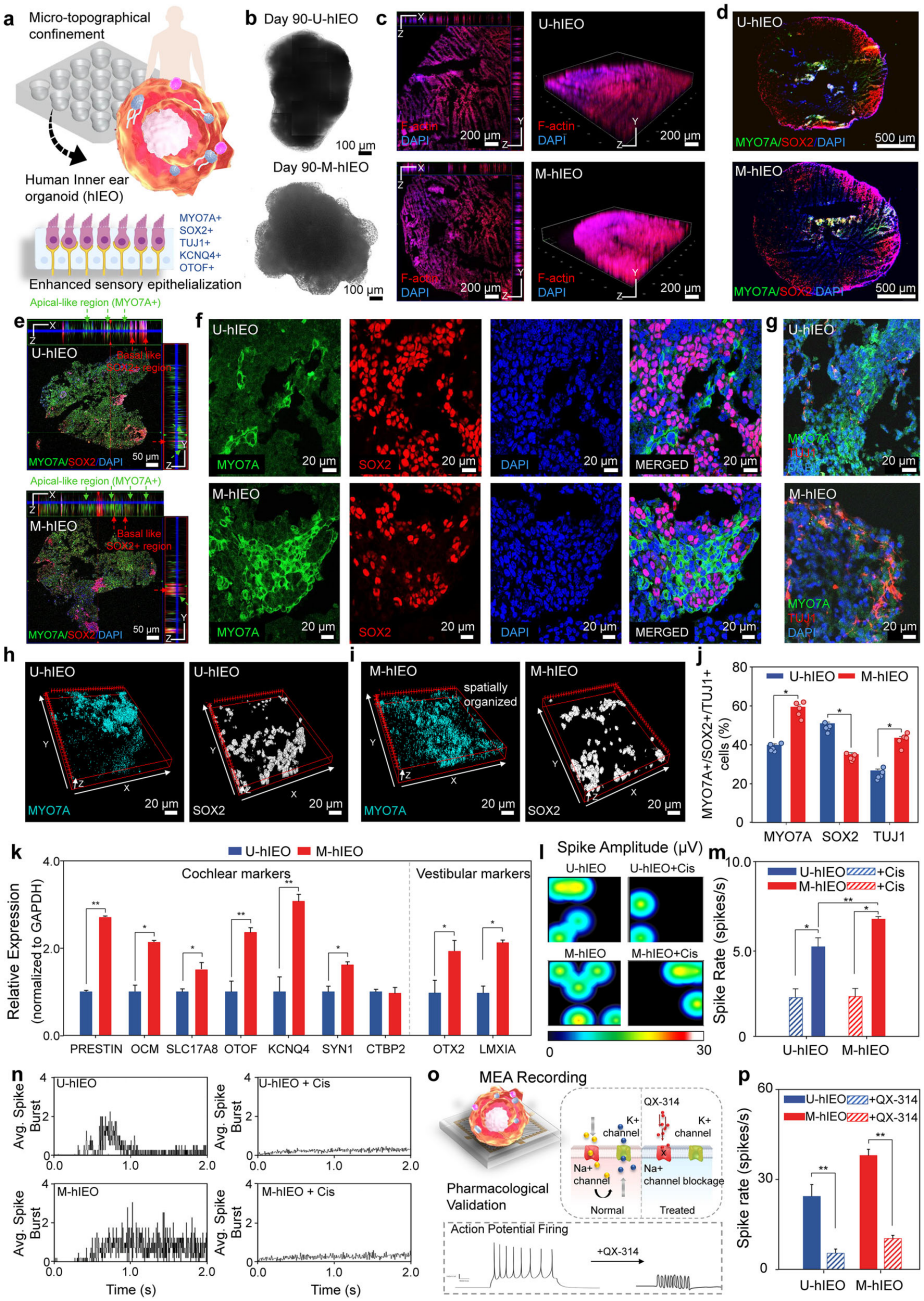
Microengineered human inner ear organoids exhibit improved structural organization and emergent electrophysiological activity. a) Schematic illustrating the generation of human induced pluripotent stem cell (hiPSC)‐derived inner ear organoids (hIEOs) under conventional U‐well (U‐hIEO) and microengineered microwell (M‐hIEO) conditions, highlighting the proposed influence of transient early micro‐topographical confinement on subsequent sensory epithelial organization. b) Representative bright‐field images of Day 90 U‐hIEOs and M‐hIEOs. Scale bars, 100 µm. c) Confocal images of Day 90 organoid sections stained with Phalloidin (F‐actin, red) and DAPI (blue), together with orthogonal (X–Z and Y–Z) views and three‐dimensional reconstruction showing overall epithelial organization. Scale bars, 200 µm. d) Low‐magnification confocal images showing the overall spatial distribution of MYO7A (green) and SOX2 (red) throughout representative Day 90 organoid sections. Scale bars, 500 µm. e) High‐magnification confocal images with orthogonal (X–Y, X–Z, and Y–Z) views illustrating the spatial relationship between MYO7A^+^ and SOX2^+^ cells. Arrows indicate the relative apical localization of MYO7A^+^ cells adjacent to the lumen and the underlying SOX2^+^ cell layer in M‐hIEOs. Scale bars, 20 µm. (f and g) Representative immunofluorescence images showing MYO7A, SOX2, and TUJ1 expression in Day 90 organoids at higher magnification. Scale bars, 20 µm. (h and i) Three‐dimensional surface‐rendered reconstructions of MYO7A^+^ and SOX2^+^ cell populations, illustrating their spatial organization within representative organoid sections. j) Quantification of MYO7A^+^, SOX2^+^, and TUJ1^+^ cell populations. k) RT‐qPCR analysis of sensory lineage‐associated genes (*MYO7A, ATOH1, ESPIN*) and additional genes associated with synaptic function, ion channel activity, and regional identity (*SLC17A8, OTOF, KCNQ4, OTX2, LMX1A*). l) Representative multi‐electrode array (MEA) activity heatmaps of U‐hIEOs and M‐hIEOs under control and cisplatin‐treated (+Cis) conditions. m) Quantification of mean spike rate derived from MEA recordings. n) Representative firing patterns and burst activity profiles, indicating increased but heterogeneous electrophysiological activity in M‐hIEOs. (o and p) Pharmacological validation using the intracellular sodium channel blocker QX‐314, showing representative recordings and quantification of spike activity before and after treatment. Data are presented as mean ± s.e.m. from n ≥ 3 independent biological replicates. Statistical tests are indicated in each panel. *p < 0.05, **p < 0.01, *p < 0.001.

To further examine the spatial distribution of sensory cell populations at Day 90, low‐magnification immunofluorescence imaging of representative organoid cross‐sections demonstrated the overall distribution of MYO7A and SOX2 across the tissue section (Figure 4d). Higher‐magnification confocal imaging followed by orthogonal (XY, XZ, and YZ) reconstruction was performed (Figure 4e). In the XY plane, MYO7A^+^ and SOX2^+^ cells were detected throughout sensory epithelial regions in both groups. Notably, orthogonal XZ and YZ views enabled visualization of their organization along the apical‐basal axis. Compared with U‐hIEOs, M‐hIEOs exhibited a more organized layered arrangement, in which MYO7A^+^ cells were predominantly localized toward the luminal surface, whereas SOX2^+^ cells occupied adjacent underlying regions. These observations are consistent with improved sensory epithelial organization and apical‐basal polarity following transient micro‐topographical confinement. To further characterize sensory epithelial organization, co‐immunostaining with the neuronal marker TUJ1 was performed. MYO7A^+^ and SOX2^+^ regions were observed in close spatial association with TUJ1^+^ neuronal projections in both organoid types (Figure 4f,g). Three‐dimensional surface‐rendered reconstructions further demonstrated that these cellular populations formed more continuous and spatially organized sensory epithelial domains throughout M‐hIEOs, whereas U‐hIEOs displayed a comparatively fragmented distribution (Figure 4h,i). Quantitative analysis confirmed a significantly higher proportion of MYO7A^+^ hair cell‐associated populations together with increased TUJ1^+^ neuronal projections in M‐hIEOs, while SOX2^+^ supporting/progenitor‐associated regions remained comparatively more prominent in U‐hIEOs (Figure 4j), consistent with enhanced sensory epithelial maturation following transient micro‐topographical confinement. Collectively, these findings indicate that early micro‐topographical confinement promotes enhanced sensory epithelial organization, enrichment of MYO7A^+^ hair cell‐associated populations, and improved three‐dimensional spatial organization of human inner ear organoids.

To further evaluate lineage‐associated maturation, RT‐qPCR analysis was performed using representative markers of sensory epithelial differentiation (Figure 4k). M‐hIEOs exhibited increased expression of sensory‐associated genes, including *MYO7A, ATOH1*, and *ESPIN*, together with genes associated with synaptic function and ion channel activity (*SLC17A8, OTOF*, and *KCNQ4*), reflecting enhanced progression toward sensory epithelial differentiation. Consistent with this interpretation, vestibular‐associated markers (*OTX2* and *LMX1A*) were detected in both groups without significant differences, indicating that both organoid populations retained mixed inner ear identities, consistent with previous reports of PSC‐derived inner ear organoids [38].

To determine whether the improved structural organization was accompanied by functional activity, spontaneous extracellular electrical activity was assessed using multi‐electrode array (MEA) recordings (Figures 4l–n). M‐hIEOs exhibited detectable spontaneous activity with significantly higher spike rates than U‐hIEOs, indicating enhanced electrophysiological activity under microengineered conditions. Upon exposure to cisplatin (15 µM, 48 h), both M‐hIEOs and U‐hIEOs exhibited reduced spike rate and burst activity (Figure 4l–n). These findings indicate that the spontaneous electrophysiological activity observed in the organoids is responsive to ototoxic perturbation. Furthermore, treatment with the intracellular sodium channel blocker QX‐314 significantly suppressed spontaneous spike activity in both groups (Figure 4o,p) supporting the contribution of sodium channel‐dependent electrical activity. However, because these recordings represent population‐level extracellular signals without single‐cell resolution or direct evidence of synaptic connectivity, they cannot be attributed to defined neuronal circuits [39]. Taken together, these findings demonstrate that transient early micro‐topographical confinement enhances the structural organization of the sensory epithelium, promotes enrichment of sensory hair cell‐associated regions, and facilitates the emergence of electrophysiologically active human inner ear organoids.

### 2.5 Transcriptomic Profiling Reveals Micro‐Topography‐Induced Mechanotransduction and Enhanced Inner Ear Developmental Programs

To elucidate the molecular basis underlying the enhanced structural organization and maturation of M‐hIEOs, transcriptomic profiling was performed on day 90 U‐hIEOs and M‐hIEOs generated under identical differentiation conditions, differing only in the initial aggregation platform. Global RNA sequencing identified 40,929 detected transcripts, including 2,508 upregulated and 2,710 downregulated genes in M‐hIEOs relative to U‐hIEOs (Figure 5a,b). To facilitate biological interpretation, representative genes associated with mechanotransduction and inner ear development were curated from the complete transcriptomic dataset based on established biological relevance together with differential expression (|log_2_ fold change| > 0.5). Consistent with this, volcano plot analysis demonstrated widespread but selective transcriptional remodeling following transient micro‐topographical confinement during the early aggregation stage (Figure 5c). Comparative analysis of global expression distributions further demonstrated an overall upward shift in transcript abundance across genes associated with mature otic lineages and sensory epithelial development in M‐hIEOs (Figure S5a,b), indicating that early topographical regulation broadly influences the transcriptional landscape of developing inner ear organoids.

**FIGURE 5:**
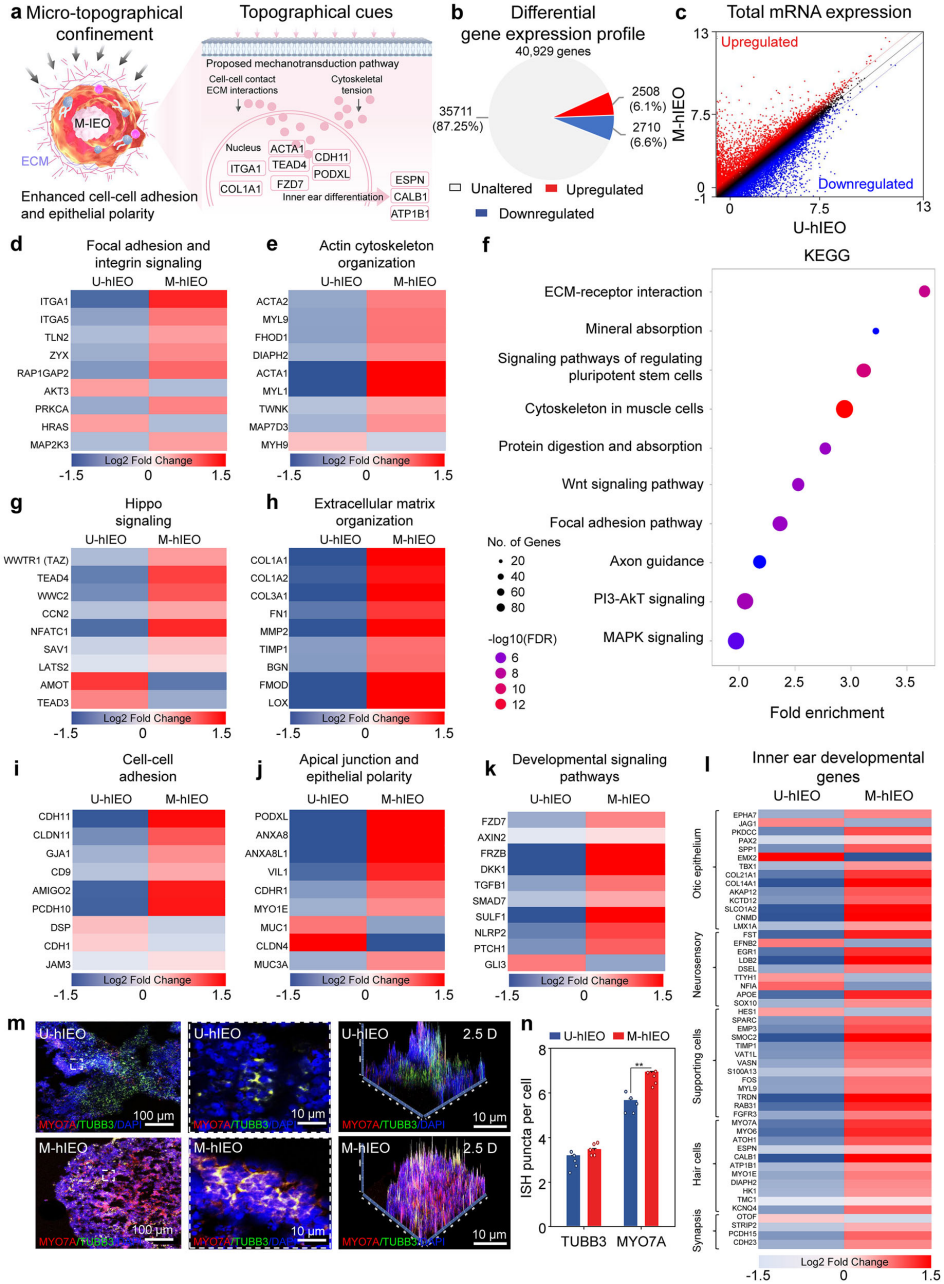
Transcriptomic profiling reveals mechanotransduction‐associated transcriptional remodeling and enhanced inner ear developmental programs induced by early micro‐topographical confinement. a) Schematic illustrating the proposed mechanism by which transient micro‐topographical confinement regulates cell‐cell adhesion, cell‐ECM interactions, cytoskeletal organization, and developmental signaling to promote enhanced maturation of microengineered inner ear organoids (M‐hIEOs). b) Distribution of differentially expressed genes identified by RNA sequencing between U‐hIEOs and M‐hIEOs. c) Volcano plot showing global transcriptional changes. (d and e) Heatmaps showing representative genes associated with focal adhesion/integrin signaling and actin cytoskeletal organization. f) KEGG pathway enrichment analysis of differentially expressed genes highlighting significantly enriched pathways related to mechanotransduction and developmental regulation, including ECM–receptor interaction, focal adhesion, WNT signaling, PI3K‐Akt signaling, MAPK signaling, and signaling pathways regulating pluripotency of stem cells. (g and h) Heatmaps showing representative genes associated with Hippo signaling and extracellular matrix organization. (i–k) Heatmaps showing representative genes involved in cell‐cell adhesion, apical junction and epithelial polarity, and developmental signaling pathways. l) Heatmap of representative genes associated with progressive stages of inner ear development, including otic epithelial specification, otic epithelial morphogenesis, neurosensory development, supporting‐cell differentiation, and hair‐cell maturation and function. m) RNAscope in situ hybridization (ISH) of MYO7A and TUBB3 in U‐hIEOs and M‐hIEOs with corresponding pseudo‐three‐dimensional fluorescence surface reconstruction. n) Quantification of MYO7A‐ and TUBB3‐positive transcript puncta per cell. Scale bars, 100 µm and 10 µm. Data are presented as mean ± s.e.m. from *n* ≥ 3 independent biological replicates. Statistical tests are indicated in each panel. *p < 0.05, **p < 0.01, ***p < 0.001.

Because transient micro‐topographical confinement primarily alters the physical microenvironment experienced during early aggregate formation, we next investigated whether these transcriptional changes were associated with remodeling of mechanotransduction‐related signaling pathways. M‐hIEOs exhibited coordinated upregulation of genes involved in focal adhesion and integrin signaling (*ITGA1, ITGA5, ZYX, AKT3*), actin cytoskeletal organization (*ACTA2, MYL9, DIAPH2, MYH9*), Hippo signaling (*WWTR1/TAZ, TEAD4, SAV1, LATS2*), extracellular matrix organization (*COL1A2, COL3A1, FN1, TIMP1, LOX*), cell‐cell adhesion (*CDH11, CLDN11, PCDH10, JAM3*), apical junction and epithelial polarity (*PODXL, CDHR1, CLDN4, MUC1*), and developmental signaling pathways including WNT, SHH, and TGF‐β signaling (*FZD7, AXIN2, FRZB, TGFB1, GLI3*) (Figure 5d–k, Table S2) [40, 41]. Collectively, these transcriptional changes suggest that transient micro‐topographical confinement is associated with coordinated remodeling of gene networks governing epithelial organization, cytoskeletal dynamics, extracellular matrix interactions, and mechanically responsive signaling, providing a molecular framework through which early geometric confinement may influence subsequent organoid maturation [42, 43].

To further evaluate whether the observed gene expression changes converged on coordinated biological processes rather than isolated transcriptional events, KEGG pathway enrichment analysis was performed using the differentially expressed genes. Enriched pathways included ECM–receptor interaction, focal adhesion, PI3K–Akt signaling, MAPK signaling, WNT signaling, and signaling pathways regulating pluripotency of stem cells (Figure 5f), further supporting the activation of mechanotransduction‐associated signaling networks following transient micro‐topographical confinement [44]. Complementary Reactome pathway enrichment analysis additionally identified enrichment of biological processes related to cell‐cycle regulation, cell differentiation, and angiogenesis (Figure S5d), indicating that early micro‐topographical confinement influences broader developmental programs beyond mechanotransduction alone.

Because mechanically responsive signaling pathways ultimately regulate developmental gene expression during organogenesis, we next examined whether the mechanotransduction‐associated transcriptional remodeling observed in M‐hIEOs was accompanied by coordinated activation of genes representing progressive stages of inner ear development. M‐hIEOs demonstrated coordinated transcriptional activation of genes involved in otic epithelial specification (*EPHA7, PKDCC, AKAP12, KCTD12*), epithelial morphogenesis (*COL21A1, COL14A1, CNMD, SLCO1A2*), neurosensory development (*APOE, FST, EFNB2, LDB2, EGR1*), supporting‐cell development (*SPARC, TIMP1, TRDN, RAB31, SMOC2*), and hair‐cell maturation and functional specialization (*ATP1B1, ESPN, CALB1, MYO1E, DIAPH2, HK1*) (Figure 5l, Table S3). Consistent with this interpretation, comparative analysis of cochlear‐ and vestibular‐associated genes (Figure S5c) demonstrated that M‐hIEOs retained transcriptional features of both sensory lineages. Although several cochlear‐associated genes, particularly *KCNQ4*, exhibited pronounced upregulation, vestibular‐associated markers were also significantly increased, indicating enhanced overall sensory maturation rather than definitive lineage specification [45].

To independently validate the transcriptomic findings using orthogonal molecular approaches, RT‐qPCR analysis was performed for selected epithelial, extracellular matrix, neuronal, and sensory markers. M‐hIEOs exhibited increased expression of genes associated with ectoderm, ECM, otic vesicles, sensory epithelium, supporting cells and hair cells (Figure S6a–g). These findings are consistent with progression toward more mature sensory epithelial states rather than prolonged maintenance of early developmental programs.

To further validate these transcriptional changes at the spatial tissue level, RNAscope in situ hybridization (ISH) was performed using MYO7A and TUBB3 as representative sensory hair‐cell and neuronal markers. M‐hIEOs exhibited stronger and more spatially organized MYO7A and TUBB3 signals than U‐hIEOs (Figure 5m). Three‐dimensional fluorescence surface reconstruction further demonstrated increased signal distribution throughout the organoid, while quantitative analysis confirmed significantly higher MYO7A‐ and TUBB3‐positive puncta per cell in M‐hIEOs (Figure 5n). Together, these findings demonstrate that transient early micro‐topographical confinement induces coordinated mechanotransduction‐associated transcriptional remodeling that is accompanied by activation of developmental gene programs and enhanced molecular maturation of PSC‐derived inner ear organoids.

### 2.6 Vascularized M‐IEO Model Enabling Vascular‐Epithelial Interactions and Inflammatory Responsiveness

To examine interactions between vascular components and inner ear organoids, we established a co‐culture system integrating M‐mIEOs with endothelial cells (ECs) and pericyte‐like cells (PCs) within a laminin‐enriched ECM (Figures 6a,b and S7). This configuration was designed to capture selected features of vascular‐epithelial organization, including EC‐PC association and extracellular matrix support, rather than to recapitulate a fully functional blood‐labyrinth barrier (BLB) [46]. Within this system, co‐culture of ECs and PCs (EC+PC+laminin) was associated with increased expression of junction‐related genes, including *CLDN1, OCLN*, and *ZO‐1*, compared to EC monoculture (Figure 6c–e). Confocal imaging revealed CD31^+^ endothelial networks in proximity to αSMA^+^ pericyte‐like cells, with heterogeneous localization of junctional proteins (Figure 6f). To further examine junctional organization within these networks, immunofluorescence staining for barrier‐associated proteins was performed. CLAUDIN‐1 and OCCLUDIN signals were detected along CD31^+^ structures, exhibiting heterogeneous and discontinuous localization patterns (Figure 6g), consistent with partial junctional organization. Merged imaging further highlighted the spatial integration of endothelial and junction‐associated signals within the network (Figure 6h). Scanning electron microscopy further indicated physical association between vascular‐like structures and the organoid surface, suggesting the formation of a spatial interface between vascular and epithelial compartments (Figure 6i,j). Functional barrier properties (permeability or transport) were not assessed.

**FIGURE 6:**
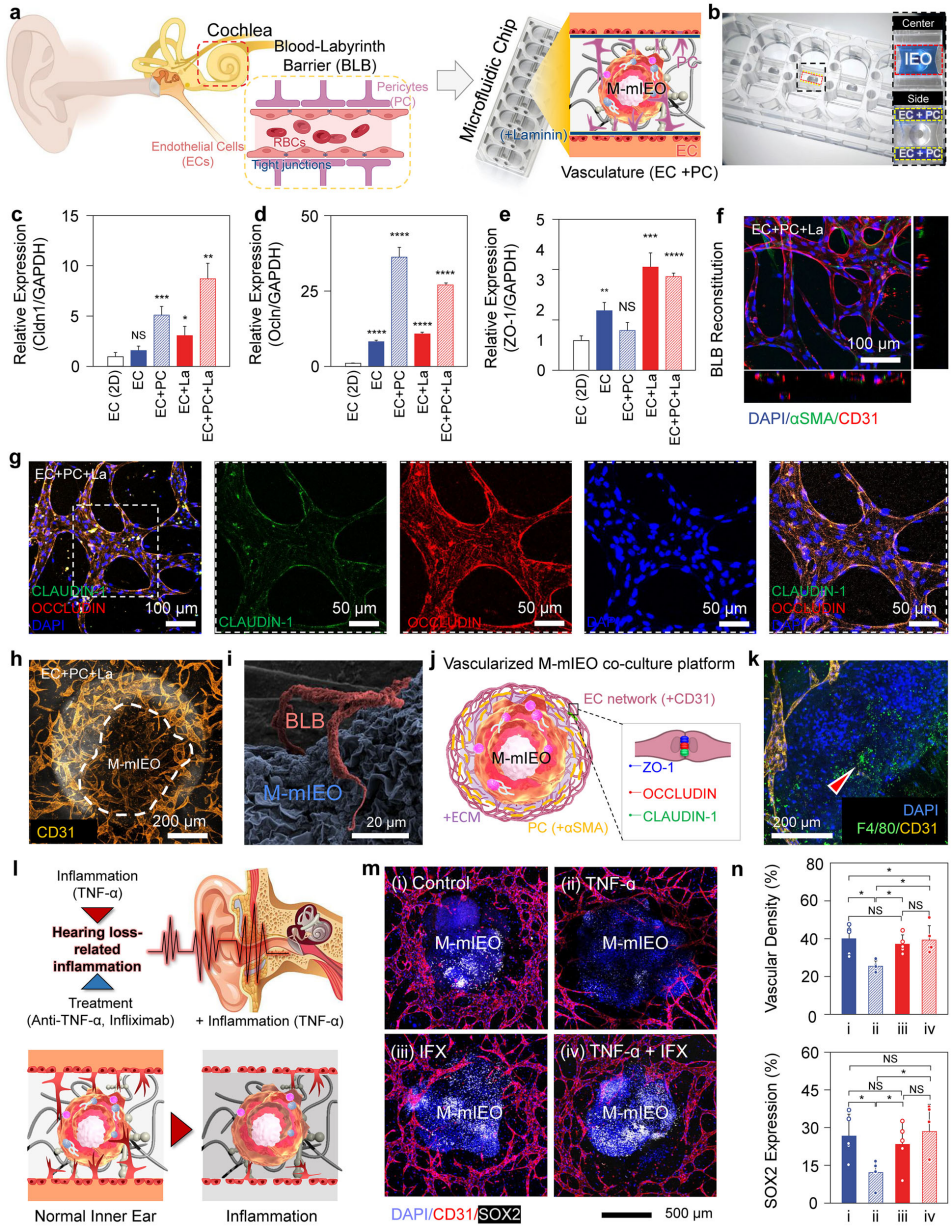
Microengineered vascular‐organoid co‐culture platform and response to inflammatory perturbation. a) Schematic illustration of the vascularized organoid‐on‐chip system integrating endothelial cells (ECs), pericyte‐like cells (PCs), and M‐hIEOs within a laminin‐enriched extracellular matrix. b) Design and layout of the microfluidic chip used for co‐culture. (c–e) RT‐qPCR analysis of junction‐associated genes (CLDN1, OCLN, ZO‐1) across culture conditions (EC alone vs EC+PC+laminin), normalized to GAPDH. f) Confocal images showing CD31^+^ endothelial networks in association with αSMA^+^ pericyte‐like cells. (g) Confocal images of junction‐associated protein expression (Claudin‐1 and Occludin), showing heterogeneous localization within vascular‐like structures. h) Higher‐magnification confocal images highlighting CD31^+^ vascular structures and perivascular organization. (i,j) Schematics and scanning electron microscopy (SEM) images illustrating physical proximity between vascular‐like structures and the organoid surface. k) Immunofluorescence staining for CD31 and F4/80. l) Schematic of inflammatory stimulation using TNF‐α and pharmacological intervention with infliximab (IFX). m) Representative images of co‐culture conditions: control, TNF‐α‐treated, IFX‐treated, and TNF‐α + IFX. n) Quantification of vascular network density and SOX2‐positive regions under indicated conditions. Data are presented as mean ± s.e.m. from *n* ≥ 3 independent biological replicates. Individual data points represent technical replicates. Statistical analysis was performed using unpaired two‐tailed Student's t‐test or one‐way ANOVA with appropriate post hoc tests, as indicated in each panel. *p < 0.05, **p < 0.01, ***p < 0.001.

To assess responsiveness to inflammatory perturbation, co‐cultures were treated with tumor necrosis factor‐α (TNF‐α; 10 ng mL^−^^1^, 24 h) (Figure 6l). Following treatment, endothelial networks exhibited reduced continuity and increased fragmentation, indicating structural sensitivity to inflammatory signaling (Figure 6m, **panel i, ii**). Concurrently, SOX2‐positive epithelial domains, used here to delineate prosensory/epithelial regions, displayed altered spatial organization. Pharmacological modulation using the TNF‐α inhibitor infliximab (IFX) resulted in partial restoration of vascular network continuity and epithelial organization (Figure 6m, **panels iii‐iv;** Figure 6n), indicating responsiveness to cytokine‐targeted intervention. These observations are based on morphological assessment; molecular and functional readouts of inflammation, including cytokine secretion or barrier permeability, were not evaluated. F4/80‐positive cells observed in the vascular co‐culture platform were derived from the mouse M‐mIEO compartment, consistent with the presence of M‐mIEO‐associated murine macrophage/resident immune‐like cells (Figure 6k). Although the vascular compartment consisted of human endothelial cells, the co‐culture enabled spatial association between M‐mIEO‐derived F4/80‐positive cells and endothelial structures, establishing a blood‐labyrinth barrier‐like vascular‐epithelial interface within the engineered platform. Collectively, these findings demonstrate the feasibility of integrating topographically engineered IEOs with a vascular microenvironment and highlight their potential for vascularized inner ear modeling and studies of inflammatory responses.

Following the establishment of mature M‐mIEOs under micro‐topographical culture conditions, we investigated whether these organoids could be integrated into a vascular co‐culture platform for blood‐labyrinth barrier‐like modeling. Mature M‐mIEOs were incorporated with endothelial and perivascular cells within a laminin‐containing microfluidic platform designed to support barrier‐associated vascular organization. The resulting co‐culture system exhibited expression of blood‐labyrinth barrier‐associated proteins, demonstrating the feasibility of integrating mature M‐mIEOs into a vascularized microphysiological platform. Accordingly, this experiment illustrates a downstream application of the mature M‐mIEO platform, extending its utility toward vascularized inner ear modeling.

## 3 Discussion

The generation of reproducible and functionally mature inner ear organoids (IEOs) remains a key challenge for developmental studies, disease modeling, and translational applications [47]. In this study, we demonstrate that transient micro‐topographical confinement during early organoid aggregation significantly enhances the structural organization, reproducibility, and functional maturation of pluripotent stem cell‐derived IEOs. These findings indicate that early biophysical cues act as instructive regulators of long‐term sensory organoid development.

Previous studies have demonstrated that micro‐topographical cues regulate cellular organization, tissue morphogenesis, and lineage specification by modulating cell‐cell and cell‐extracellular matrix interactions within the developing microenvironment [18]. However, microwell‐based platforms have been primarily utilized to facilitate the formation of embryoid bodies, spheroids, and early organoid aggregates. They have primarily served as culture tools to improve aggregate formation, while the biological consequences of early micro‐topographical regulation on subsequent organoid development have remained largely unexplored [48]. To our knowledge, this study provides the first systematic investigation demonstrating that transient micro‐topographical regulation during early organoid morphogenesis durably influences the structural and functional development of PSC‐derived inner ear organoids. In this study, we introduce a transient micro‐topographical engineering strategy in which temporally defined microscale confinement is applied during the early stages of IEO differentiation. Notably, our findings demonstrate that both the timing and duration of micro‐topographical cues are critical determinants of subsequent M‐IEO development and maturation [49, 50].

Interestingly, transient confinement (5 days) yields more structurally intact organoids than prolonged confinement (10 days), highlighting the importance of temporal control in guiding early morphogenesis. Previous studies suggest that this effect may reflect a balance between spatially coordinated cell–cell assembly during early aggregation and the need for unrestricted tissue growth during subsequent morphogenesis. Prolonged confinement may impose diffusion‐related constraints and limit tissue remodeling, whereas transient confinement establishes early structural organization while preserving the developmental plasticity required for continued growth and maturation [6, 51, 52, 53, 54]. Collectively, these findings suggest that transient micro‐topographical guidance provides an instructive biophysical environment for early tissue organization, followed by release into suspension culture that supports continued tissue expansion, remodeling, and functional maturation. Importantly, these effects cannot be attributed solely to differences in aggregate size or compaction but instead reflect early geometry‐driven mechanobiological regulation that establishes persistent developmental programming during subsequent organoid maturation.

Mechanistically, transient micro‐topographical confinement likely regulates early mechanotransduction through integrin‐mediated cell‐ECM interactions and cytoskeletal remodeling, thereby influencing downstream developmental signaling pathways [55]. Consistent with this interpretation, transcriptomic profiling of day 90 M‐hIEOs revealed coordinated upregulation of mechanotransduction‐associated gene networks, including focal adhesion and integrin signaling (*ITGA1, ITGA5*), actin cytoskeletal remodeling (*MYH9, DIAPH2*), Hippo signaling (*WWTR1/TAZ, TEAD4*), extracellular matrix organization (*FN1, COL1A2*), cell‐cell adhesion (*CDH11, JAM3*), and apical junction/polarity‐associated genes (*PODXL, CDHR1*) [56]. KEGG pathway enrichment analysis further identified enrichment of ECM‐receptor interaction, focal adhesion, PI3K‐Akt, MAPK, and WNT signaling pathways as major pathways associated with the microengineered condition. Recent studies further suggest that tissue mechanics, cytoskeletal tension, and cell‐ECM interactions are closely integrated with these developmental signaling networks [57]. Specifically, Moore et al., demonstrated that SHH signaling is necessary to ventralize the otocyst and derive cochlear structures whereas WNT signaling pathway has been shown to play a role in dorsal gene expression (vestibular) during inner ear development [58, 59, 60]. Consistent with this framework, the enrichment of WNT‐ and SHH‐associated genes (*SULF1, LRP2, PTCH1, FZD7, AXIN2*) observed in M‐hIEOs suggests that transient micro‐topographical confinement may promote inner ear developmental progression by coupling mechanically responsive signaling with canonical morphogenetic programs. Notably, because M‐hIEOs and U‐hIEOs were cultured identically after early aggregation, these sustained transcriptional differences indicate that micro‐topographical cues induce durable developmental reprogramming. Although the present transcriptomic analyses provide evidence for coordinated mechanotransduction‐associated remodeling, direct perturbation of these pathways will be required to establish causal mechanisms. Nevertheless, our findings support establishment of a mechanically instructive microenvironment that initiates persistent mechanotransduction‐associated transcriptional remodeling of PSC‐derived inner ear organoids [61, 62].

Additionally, M‐hIEOs exhibited enhanced sensory epithelial organization compared with U‐hIEOs. High‐resolution orthogonal confocal imaging demonstrated a more organized spatial arrangement of MYO7A^+^ and SOX2^+^ cell populations, with MYO7A^+^ cells preferentially localized toward the luminal (apical) surface and SOX2^+^ cells positioned beneath this layer within localized sensory epithelial regions. Complementary 3D surface reconstruction demonstrated larger, more continuous, and spatially organized sensory epithelial domains containing MYO7A^+^ and SOX2^+^ cell populations in M‐hIEOs compared with U‐hIEOs. These structural observations were further supported by transcriptomic enrichment of genes associated with apical junction organization, epithelial polarity and sensory‐associated genes, including *MYO7A, ATOH1, OCM, ESPN, PRESTIN*, and *SLC17A8*, together with the presence of F‐actin‐rich structures [63]. Beyond epithelial organization, M‐IEOs also exhibited features of progressive neurosensory maturation with enhanced MYO7A‐positive sensory hair cell‐like populations and TUJ1‐positive neuronal networks and synaptic function (*SLC17A8, SYN1*, and *CTBP2*). Nevertheless, although orthogonal imaging, three‐dimensional reconstruction, and transcriptomic analyses collectively support improved sensory epithelial organization, future studies incorporating single‐cell transcriptomic profiling together with additional polarity and synaptic markers will be important to resolve cellular heterogeneity and further define the molecular identity and maturation states of individual cell populations, as well as to determine the extent to which micro‐topographical confinement promotes native‐like sensory epithelial organization within the organoids.

Functionally, FM1‐43 uptake is widely used as an indirect readout of mechanosensitive hair cell‐like activity, reflecting entry through mechanotransduction channels as well as endocytic membrane uptake pathways in sensory epithelial cells [64]. M‐IEOs exhibited increased FM1‐43 uptake, suggesting an enhanced presence and broader distribution of FM1‐43‐permeable mechanosensitive hair cell‐like populations. Importantly, the observed labeling was cell‐associated rather than diffuse, supporting its interpretation as a functional uptake signal. In addition, phalloidin staining revealed prominent F‐actin‐rich stereocilia‐like structures, while TEM identified kinocilium‐like apical structures exhibiting a characteristic (9 × 2) +2 microtubule organization. Collectively, these findings further support enhanced structural and functional maturation of sensory epithelial domains following transient micro‐topographical regulation.

Importantly, previous landmark studies on stem cell‐derived inner ear organoids have demonstrated that electrophysiological maturation occurs in a gradual and heterogeneous manner. For example, Koehler et al. reported that ESC‐derived hair cell‐like cells exhibit voltage‐dependent potassium currents and mechanotransduction‐associated activity within the picoampere to sub‐nanoampere range that progressively increase during in vitro maturation but remain below those of fully mature native hair cells [35]. Likewise, previous studies using ESC‐ and iPSC‐derived hair cell‐like cells have consistently reported voltage‐dependent potassium and inward currents within the picoampere range, supporting the concept that electrophysiological maturation develops progressively during stem cell‐derived inner ear differentiation [65, 66]. Consistent with these observations, the voltage‐dependent outward currents detected in M‐IEO‐derived cells are indicative of early electrophysiological maturation of hair cell‐like populations within the microengineered organoids. Although mechanotransduction currents, the gold‐standard functional hallmark of mature hair cells, were not evaluated in the present study, the electrophysiological findings provide supportive functional evidence when interpreted together with the enhanced sensory epithelial organization, ultrastructural characterization, transcriptomic analyses, and complementary functional assays presented throughout this study. Future studies incorporating direct mechanotransduction measurements and larger electrophysiological datasets across independent organoid preparations will further strengthen the functional characterization of these cells.

Complementarily, MEA recordings demonstrated enhanced spontaneous electrophysiological activity in M‐IEOs compared with U‐IEOs, indicating increased functional excitability within the organoid microenvironment. Pharmacological suppression of this activity by QX‐314, a voltage‐gated sodium channel blocker, confirmed that the observed signals are predominantly mediated by Na^+^ channel‐dependent mechanisms, consistent with biologically active excitable cell populations [67]. Together with the increased expression of synaptic and neurodevelopmental genes, these findings suggest an overall advancement in the functional maturation state of neurosensory‐like components within M‐IEOs, although they do not by themselves establish fully organized neuronal network activity or mature sensory circuitry.

To further examine whether transient micro‐topographical confinement biased sensory lineage specification, we compared the expression of representative cochlear‐ and vestibular‐associated genes based on established inner ear organoid datasets. M‐mIEOs showed increased expression of several cochlear‐associated genes, including *TMC1, KCNQ4, OTOF, STRIP2*, and *ATP1B1* however, vestibular‐ and pan‐sensory markers, including *CALB1, MYO7A, MYO6, ESPN*, and *PCDH15*, were also retained. Overall, while micro‐topographical confinement enhances sensory epithelial maturation, it does not appear to selectively drive cochlear versus vestibular specification under the present conditions. Instead, the biophysical microenvironment likely improves overall developmental competence, generating a permissive state that may later be refined by spatiotemporally controlled morphogen signaling (WNT, SHH, BMP, or FGF pathways) to achieve subtype‐specific patterning [45]. Future single‐cell transcriptomic and polarity‐based analyses will be required to further define the extent of cochlear vs. vestibular lineage commitment induced by micro‐topographical regulation.

An important outcome of transient micro‐topographical regulation is the marked reduction in organoid‐to‐organoid variability. Conventional organoid systems often exhibit heterogeneity in size, morphology, and differentiation efficiency, limiting their reproducibility and scalability. In contrast, controlled early aggregation produced more uniform organoids with consistent structural and transcriptional features. Notably, these effects were observed across both mouse and human systems, suggesting that the influence of early topographical cues represents a conserved principle in pluripotent stem cell‐derived organoid development.

Beyond M‐IEO development, we extend this framework to a microengineered vascularized co‐culture system that enables controlled interaction between M‐IEOs and vascular components. The vascular co‐culture experiment demonstrates a potential application of the mature M‐mIEOs established in this study by capturing selected structural aspects of vascular‐epithelial organization and showing responsiveness to inflammatory perturbation and pharmacological modulation. Specifically, incorporation of mature M‐mIEOs into a vascular co‐culture platform was associated with the expression of blood‐labyrinth barrier function‐related markers, suggesting that matured IEOs may provide a suitable organoid component for constructing a blood‐labyrinth barrier‐like microphysiological model. However, the current study assessed marker expression rather than direct functional barrier properties. Therefore, this platform should be interpreted as an initial blood–labyrinth barrier‐like model showing molecular features related to barrier function. Further validation of barrier function will require functional validation, such as permeability assays, trans‐endothelial electrical resistance measurements, or selective transport analyses, to confirm blood‐labyrinth barrier function more rigorously. This platform provides a foundation for future studies incorporating immune components and functional barrier assays [68, 69, 70, 71, 72]. Another limitation of the current model is the cross‐species nature of the vascular co‐culture, in which mouse M‐mIEOs were cultured with human HUVECs and HBVPs. Although previous studies have demonstrated the feasibility of using HUVEC‐based vascular compartments to support organoid culture, including mouse organoid systems, this configuration does not fully recapitulate a species‐matched or inner‐ear‐specific vascular niche [73, 74, 75]. Thus, the present system should be interpreted as an engineered vascular‐support platform rather than a fully physiological developmental model.

Overall, these findings demonstrate that early topographical cues act as instructive regulators of inner ear organoid development, influencing structural organization, reproducibility, and functional progression. More broadly, this work highlights that transient control of the early microenvironment can have lasting effects on tissue development, providing a useful framework for improving the consistency and physiological relevance of organoid systems and advancing neurosensory research, disease modeling, and therapeutic screening.

## 4 Conclusion

We demonstrate that transient micro‐topographical engineering provides an instructive biophysical strategy for directing PSC‐derived inner ear organoid development. Early micro‐topographical cues act as instructive microenvironmental signals that durably influence subsequent organoid morphogenesis and developmental progression. This temporally restricted microscale confinement enhances reproducibility, improves structural organization, and promotes coordinated molecular and functional maturation across both mouse‐ and human‐derived inner ear organoids. Furthermore, integration of these microengineered organoids with vascular components establishes a controllable vascularized platform for investigating vascular‐epithelial interactions and inflammatory responses. Collectively, these findings establish transient micro‐topographical regulation as a practical engineering strategy for improving inner ear organoid reproducibility and maturation, providing a robust platform for disease modeling, mechanistic studies, and therapeutic screening.

## 5 Experimental Section/Methods

### 5.1 Fabrication of Microwell Chips

Microwell chips were fabricated using a soft lithography‐based replica molding approach to generate a geometrically defined micro‐topographical culture platform for controlled early‐stage organoid aggregation. The microwell architecture was designed to provide uniform hemispherical confinement, enabling reproducible control over initial organoid development. Briefly, a pre‐patterned master mold containing an array of hemispherical microwells (500 µm diameter, 225 wells per unit chip) was used to define the microscale geometry of the culture platform. Poly (urethane acrylate) (PUA; 301RM, Minuta Tech, Republic of Korea) precursor solution was poured onto the mold and subjected to vacuum to ensure complete filling of the microwell structures and removal of trapped air bubbles. The PUA was then photopolymerized under UV irradiation (*λ* = 352 nm, 40 W) for 3 h and carefully peeled off to generate a negative replica mold preserving the defined micro‐geometric features. Polydimethylsiloxane (PDMS; Sylgard 184 Silicone Elastomer Kit, Dow Corning, USA) was prepared by mixing base and curing agent at a ratio of 10:1 (w/w), followed by vacuum degassing to eliminate residual air bubbles. Approximately 30 g of the PDMS mixture was cast onto the PUA replica mold and thermally cured at 100°C for 4 h. After curing, the PDMS layer was gently detached to obtain the final microwell chip, which preserved the uniform hemispherical micro‐topography. Each fabricated chip contained 225 microwells and was placed into a standard 6‐well plate format (one chip per well) for subsequent cell seeding, aggregate formation, and organoid differentiation.

### 5.2 Pluripotent Stem Cell (PSC) Culture

Human induced pluripotent stem cells (hiPSC, hFSiPS3‐1, passages 35–50, Korea National Institute of Health, Republic of Korea) were cultured in mTeSR1 medium (Stem Cell Technologies, #85850) supplemented with 10 µM ROCK inhibitor (Y‐27632 dihydrochloride, Tocris Bioscience, #1254) on 1% Matrigel (Corning, hESC‐qualified, #354277) coated 6‐well plates prepared in DMEM/F12 (Gibco, #11330‐032), according to the established protocol. Cells were sub‐cultured every 4 days at approximately 70% confluency using Accutase containing 0.5 mM EDTA (Stem Cell Technologies, #07920) at a split ratio of 1:10.

Mouse embryonic stem cells (mESCs, R1/E, passages 10‐30, SCRC‐1036, American Type Culture Collection, ATCC, Manassas, VA, USA) were cultured and maintained in LIF‐2i medium. For 50 mL of medium, 41.44 mL of Advanced DMEM/F12 (Gibco, #12634010) supplemented with 15% fetal bovine serum (FBS) (Gibco, #12483020), 500 µl 1 x MEM non‐essential amino acids (NEAA) (Gibco, #11140050), 500 µL of 100× GlutaMAX Supplement (Gibco, #35050‐061), 5 µL of mouse leukemia inhibitory factor (mLIF, Merck, #ESG1107), 1.65 µL of CHIR‐99021 (Stemolecule, #04‐0004‐02), 0.25 µL of PD035901 (Stemolecule, #04‐0006‐02, CA), 0.34 µL of 2‐mercaptoethanol (Sigma‐Aldrich, #M6250), and 50 µL of penicillin‐streptomycin (Sigma‐Aldrich, #P4333) were combined. mESC culture and maintenance were performed according to the previously reported protocol by Koehler et al. At approximately 80% confluency (∼2 d), mESCs were passaged at a 1:10 split ratio. On day 2, cells were washed with 1× PBS and treated with 0.25% trypsin‐EDTA (Gibco, #25200056), and cell clumps were dissociated by gentle pipetting without introducing air bubbles.

### 5.3 Generation of Inner Ear Organoids (IEOs)

Human or mouse pluripotent stem cells (hiPSCs/mESCs) were aggregated and differentiated into inner ear organoids following a previously established protocol. Organoids generated in conventional U‐well plates are referred to as U‐mIEOs/U‐hIEOs, while those formed using microengineered PDMS microwell chips are referred to as M‐mIEOs/M‐hIEOs. The PDMS microwell platform provides defined microscale confinement enabling controlled aggregation without external force‐driven compaction. Both groups were subjected to identical differentiation conditions (cell number (4 x 10^3^), medium composition, growth factors, and timing), with the culture platform geometry representing the only independent experimental variable.

Cells were dissociated into single cells, counted, and seeded at a density of 4 × 10^3^ cells per well in both the platforms. For microwell chips, cells were added in 150 µL of ectoderm differentiation medium (mouse) or mTeSR1 medium (human) per chip, whereas 100 µL per well was used for U‐well plates. Medium volumes were adjusted according to the physical requirements of each culture format to ensure full coverage of the culture area and do not constitute an experimental variable. The differentiation medium consisted of GMEM (Gibco, #11710‐035) supplemented with 15% KnockOut Serum Replacement (Gibco, #10828028), sodium pyruvate (Gibco, #11360070), β‐mercaptoethanol (Sigma‐Aldrich, #M6250), and 1% penicillin‐streptomycin (Sigma‐Aldrich, #P4333). For hiPSC‐derived organoids, the medium was additionally supplemented with 10 µM ROCK inhibitor and 1 × MEM Non‐Essential Amino Acids (NEAA). Following seeding, microwell chips were incubated overnight at 37°C in 5% CO_2_ to allow cells to settle into the microwells. On day 1, differentiation medium supplemented with 2% Matrigel (final concentration) was added. For the microengineered condition, each PDMS microwell chip (containing 225 individual microwells) was cultured in one well of a 6‐well plate containing 5 mL of differentiation medium. In the conventional U‐bottom condition, each well received 100 µL of differentiation medium containing the same final concentration (2%) of Matrigel. On day 3, differentiation medium (1.25 mL per chip and 25 µl per well) containing BMP4 and SB431542 (added at 5× concentration) was introduced, resulting in final concentrations of 10 ng/mL BMP4 and 1 µM SB431542, and cultures were maintained for an additional 2 days. On day 5, FGF2 and LDN‐193189 (added at 6× concentration) were introduced (1.25 mL per chip and 25 µl per well), yielding final concentrations of 25 ng/mL FGF2 and 1 µM LDN‐193189. The total medium volume per chip was 7.5 mL, and cultures were maintained for 3 days. On day 8, organoids formed in microwell chips were harvested and transferred to ultra‐low attachment 24‐well plates. At this stage, complete medium was replaced with maturation medium consisting of Advanced DMEM/F12 supplemented with N2 supplement, 1% Matrigel, and 3 µM CHIR99021 for 2 days. From day 10 onward, organoids were maintained in maturation medium with media changes every other day for 30 days (mIEOs) or 90 days (hIEOs). Importantly, no centrifugation was applied during aggregation in either U‐bottom plates or microwell chips, ensuring that all observed differences arise solely from culture geometry rather than handling variations.

### 5.4 FM1‐43 Uptake Assay

To assess functional mechanotransduction activity in hair cell‐like populations, FM1‐43 dye uptake was performed on live inner ear organoids. Mature organoids were incubated with FM1‐43 dye (Invitrogen, FM 1‐43, #T3163) prepared in culture medium at working concentration (5 µM). Organoids from both control conditions and cisplatin‐treated groups were incubated with DMEM/F12 medium for pre‐equilibration followed by incubation with the dye for 1 min at 37°C under standard culture conditions. Following incubation, organoids were gently washed three times with ice cold PBS to remove excess extracellular dye. Live imaging was performed immediately after washing using a fluorescence microscope to visualize dye uptake.

### 5.5 Patch Clamp Electrophysiology

Whole‐cell patch‐clamp recordings were performed in voltage‐clamp mode at room temperature using standard borosilicate glass electrodes. Voltage protocols consisted of 10 mV incremental steps from a holding potential of ‐84 mV to test potentials ranging from ‐124 to +54 mV for 170 ms, followed by repolarization to ‐44 mV to record tail currents (50 ms). The extracellular bath solution consisted of Leibovitz's L‐15 medium (Gibco). Patch pipettes were filled with an intracellular solution containing 150 mM KCl, 5 mM HEPES, 0.1 mM CaCl_2_, 5 mM EGTA, and 3.5 mM Mg‐ATP (pH 7.3 adjusted with KOH; osmolality 288 mmol/kg). Pipette resistance ranged from 3‐6 MΩ. For electrophysiological recordings, day 90 inner ear organoids were gently dissociated into single cells using brief trypsin treatment (5 min) followed by mechanical trituration. Cells were allowed to settle prior to recording. Cells selected for patch‐clamp analysis were chosen based on epithelial morphology, appropriate cell size comparable to cochlear hair cells, and stable membrane properties, including high input resistance and characteristic voltage‐dependent ionic currents. Electrophysiological properties of organoid‐derived cells were directly compared with those of freshly isolated rat cochlear hair cells recorded under identical experimental conditions to provide functional reference benchmarks. A total of *n* = 4 cells was recorded per condition.

### 5.6 Multi‐Electrode Array (MEA) Recording

Mature IEOs from both groups were harvested and transferred to 1% Matrigel‐coated 24‐well MEA plates (Axion BioCircuit MEA, Axion Biosystems; 16 gold electrodes per well, #M384‐BIO‐24) and incubated for 4 days prior to recording to allow stable attachment. One organoid was seeded per well; therefore, each well represented one independent biological replicate (*n* ≥ 3 organoids per condition), while the 16 electrodes within each well served as technical recording channels to capture spatially distributed electrophysiological activity from the same organoid. Spike recordings were acquired using an Axion Maestro system controlled by AxIS Navigator software version 2.4 (Axion Biosystems, Atlanta, GA, USA) at a sampling rate of 12.5 kHz. Hardware settings were configured with 1200× gain and a bandwidth of 10‐5000 Hz. Organoids were maintained at 37°C and 5% CO_2_ during recording, and spontaneous activity was recorded for 10 min every 5 min. Raw MEA data were processed using Neural Metric software. Spike detection was performed in MATLAB, and detected spike times were assigned to their corresponding electrodes and exported as.csv files. Bursts were identified using a modified logISI algorithm with a minimum threshold of five spikes, after which burst‐associated parameters were calculated. For quantitative analysis, spike amplitude, spike rate, and burst‐related parameters were first calculated at the individual electrode level. All electrodes were included in the initial recordings; however, electrodes that did not exhibit detectable activity throughout the recording period were classified as inactive channels and were not included in average calculations at the organoid level to avoid distortion from non‐informative signals. The resulting per‐well averaged values were used as a single biological replicate for statistical analysis.

### 5.7 QX‐314 Treatment (Electrophysiology Modulation)

To evaluate the contribution of ion channel‐mediated activity in inner ear organoids, pharmacological inhibition was performed using QX‐314 (Calbiochem, CAS 21306‐56‐9, #552233‐100MGCN). Mature organoids were incubated with QX‐314 prepared in culture medium in MEA plates at working concentration (100 µM) for 1 h at 37°C under standard culture conditions. Following treatment, organoids were directly transferred to multi‐electrode array (MEA) plates for electrophysiological recording. Spontaneous electrical activity was recorded using the MEA system as described above. Changes in spike frequency and burst activity following QX‐314 treatment were analyzed to assess the contribution of ion channel activity to the observed electrophysiological signals.

### 5.8 Organoid Processing and Cryosectioning

Organoids were harvested at designated differentiation time points, gently washed with PBS, and fixed in 4% paraformaldehyde (PFA) at 4°C for 30–60 min, depending on organoid size (1–2 mm), followed by three washes with PBS. Fixed organoids were cryoprotected by sequential incubation in 15% and 30% (w/v) sucrose solutions in PBS at 4°C until the samples sank, indicating complete cryoprotection. Cryoprotected organoids were embedded in optimal cutting temperature compound (Sakura Finetek, Tissue‐Tek O.C.T. Compound, #4538, Japan) in cryomolds and rapidly frozen at ‐20°C, then stored at ‐80°C until sectioning. Cryosections (10 µm thickness) were prepared using a cryostat (Leica Biosystems, Leica CM1860, Germany) and mounted onto positively charged glass slides (Paul Marienfeld GmbH & Co., Histobond adhesive slides, #0810001, Germany). Sections were air‐dried at room temperature, stored at ‐20°C, and subsequently used for immunofluorescence staining according to experimental requirements.

### 5.9 Immunofluorescence Staining

Cryosections or whole‐mount organoids on day 3 (ectoderm), day 15 (otic vesicles) and day 30/90 (hair cells and supporting cells maturation) were permeabilized with 0.1% Triton X‐100 in PBS for 30 min and washed three times with 1× PBS for 5 min each. Samples were then blocked with 1% bovine serum albumin (BSA; Cell Signaling Technology, #9998S) for 3 h, followed by incubation with primary antibodies diluted in BSA for 24 h. Primary antibodies were anti‐MYO7A (Proteus Biosciences Inc., #25‐6790), anti‐SOX2 (Cell Signaling Technology, #4900), beta‐3 Tubulin Monoclonal Antibody (2G10) (Invitrogen, #MA1‐118), anti‐laminin β1 (Abcam, #ab44941), anti‐Pax8 (Abcam, #ab53490), and E‐cadherin (Cell Signaling Technology, #3195). After washing three times with 1x PBS, organoids were stained with PBS‐diluted secondary antibodies (Jackson ImmunoResearch, Cy3 AffiniPure Goat Anti‐Mouse IgG (H+L), #115‐165‐003; Jackson ImmunoResearch, Fluorescein (FITC) AffiniPure Goat Anti‐Rabbit IgG (H+L), #111‐095‐003). After washing three times with 1x PBS for 5 min each time, the nuclei of the cells were stained with diluted 4,6‐diamidino‐2‐phenylindole (DAPI, Thermo Fisher Scientific, #D1306). Furthermore, organoids were mounted using ProLong Gold antifade reagent (Invitrogen, #P36935). Fluorescence images were acquired using a laser scanning confocal microscope system. Relative fluorescence intensity was quantified using ImageJ software.

For vascularized IEOs, immunofluorescence imaging was performed to visualize cell‐specific marker expression. The samples were fixed with 4% (w/v) PFA for 15 min, followed by permeabilization in 0.1% Triton X‐100 for 15 min. The samples were then treated with 3% BSA for 40 min. The following antibodies were used for immunocytochemistry and were incubated for two days at 4°C: AlexaFluor 594 conjugated mouse anti‐CD31 (1:200; BioLegend), AlexaFluor 647 conjugated mouse anti‐CD31 (1:200; BioLegend), AlexaFluor 488 conjugated mouse anti‐α‐ SMA (1:200; R&D systems), AlexaFluor 488 conjugated mouse anti‐claudin 1 (1:200; Invitrogen), AlexaFluor 488 conjugated rat anti‐F4/80 (1:200; Invitrogen), AlexaFluor 594 conjugated mouse anti‐occludin (1:200; Invitrogen), AlexaFluor 647 conjugated mouse anti‐ ZO‐1 (1:200; Invitrogen), AlexaFluor 660 conjugated rat anti‐SOX2 (1:200; Invitrogen), and Hoechst 33342 (1:1,000; Invitrogen). Fluorescence images were acquired using a laser scanning confocal microscope system. Relative fluorescence intensity was quantified using ImageJ software. Vascular density was quantified using ImageJ software (v1.53). Briefly, maximum intensity projections of CD31‐stained images were thresholded to create binary masks, and the “Area Fraction” was calculated as the percentage of CD31‐positive pixels within the total defined ROI of the organoid.

### 5.10 In situ Hybridization (ISH)

In situ hybridization was performed using the RNAscope Multiplex Fluorescent Reagent Kit v2 (ACD Bio‐Techne, #323100) according to the manufacturer's instructions. Inner ear organoids were fixed in 4% paraformaldehyde, washed with phosphate‐buffered saline (PBS), and processed for probe hybridization following standard RNAscope protocols. Samples were subjected to target retrieval and protease treatment prior to probe hybridization. The following target‐specific probes were used: Hs‐MYO7A (#846251) and Hs‐TUBB3‐C2 (#318901‐C2). Probes were diluted using RNAscope Probe Diluent (#300041) and hybridized to the samples under conditions specified by the manufacturer. A 3‐plex positive control probe (Hs, #320861) was used to validate assay performance. Signal amplification was carried out using the provided amplification reagents, followed by detection using Opal fluorophores (Opal 520 Reagent Pack, #FP1487001KT; Opal 570 Reagent Pack, #FP1488001KT). Fluorescent signals were visualized using a confocal microscope.

### 5.11 Confocal microscopy

Images were taken using a ZEISS LSM 900 in confocal mode, using Zen 3.1. Fluorescence readouts were obtained at an excitation and emission of 488 nm and 507 nm for green and 594 nm, 614 nm for red and 358 nm and 405 nm for blue. Laser power, gain and pinhole settings were adjusted, and Z stack images were constructed. Acquired images were processed using ZEISS ZEN microscopy software.

### 5.12 Scanning Electron Microscopy (SEM)

To characterize the microarchitecture of the fabricated devices, microwell chips were first imaged using scanning electron microscopy (SEM). Briefly, PDMS microwell chips were cut into appropriate sizes, mounted onto aluminum stubs using conductive carbon tape, and sputter‐coated with a thin layer of platinum (∼5 nm). Imaging was performed using a field‐emission scanning electron microscope to confirm microwell geometry, diameter, and surface morphology.

For biological samples, inner ear organoids at defined time points (days 1, 3, and 30) were obtained using a JSM‐7500F field emission scanning electron microscope (JEOL Ltd., Tokyo, Japan). In brief, aggregates were fixed with 2% paraformaldehyde (Sigma‐Aldrich) and 2% glutaraldehyde (Sigma‐Aldrich) in 0.05 M PBS (Sigma‐Aldrich) for 4 h. The samples were then washed three times with 0.05 M PBS and finally fixed with 1% osmium tetroxide (Sigma‐Aldrich) for 3 h. The samples were then washed three times with 1%PBS for 10 min each time and dehydrated with a graded concentration of ethanol (30%, 50%, 70%, 90%, and 100%, v/v). The samples were finally coated with a Pt layer (∼5 nm thick) by metal sputtering, and SEM images were obtained.

For vascularized IEOs, the constructs were washed in PBS, then fixed in a 2% glutaraldehyde solution for 2 h. The constructs were washed in 0.05 M sodium cacodylate buffer (Sigma‐Aldrich) for 10 min, and the washing step was repeated twice. Constructs were fixed with 1% osmium tetroxide (Sigma‐Aldrich) for 1 h at 4°C. Then, fixed samples were dehydrated with a series of 30%, 50%, 70%, 80%, 90%, and 100% (× 3) ethanol solutions for 10 min each. Subsequently, dehydrated samples were frozen for 3 h at ‐80°C and then air dried for 24 h in a vacuum desiccator. The dried samples on the adhesive film were prepared by removing the microfluidic chip body from the film. Finally, the samples were mounted on aluminum stubs, sputter‐coated with platinum, and examined by FE‐SEM (SIGMA, Carl Zeiss).

### 5.13 Transmission Electron Microscopy (TEM)

For transmission electron microscopy (TEM), day 30 inner ear organoids were fixed and processed following standard protocols. Ultrastructural analysis was targeted at organizing epithelial regions lining lumen‐like structures within the organoids. Regions of interest were selected based on prior immunofluorescence characterization of parallel samples, which identified Myo7a‐positive sensory epithelial domains. Cells located at the apical surface facing the lumen were examined and classified as hair cell‐like based on established ultrastructural hallmarks, including the presence of a kinocilium with a 9+2 axonemal microtubule arrangement, an underlying cuticular plate, and actin‐rich apical protrusions consistent with stereociliary bundle‐like structures.

### 5.14 Quantitative Real Time‐PCR

For all RT‐qPCR analyses, RNA was isolated from entire organoids, and therefore gene expression reflects whole‐organoid transcriptional responses rather than cell‐type‐specific changes. Total RNA was extracted using TRIzol Reagent (Invitrogen, #15596026, USA) according to the manufacturer's instructions. RNA concentration and purity were assessed using NanoDrop Lite spectrophotometer (Thermo Scientific, USA). Complementary DNA (cDNA) was synthesized from 2 µg of total RNA using the GoScript Reverse Transcription System (Promega, #A501D, USA) following the manufacturer's protocol. Quantitative real‐time PCR was performed using GoTaq qPCR Master Mix (2X) (Promega, #A6001, USA) on a Bio‐Rad real‐time PCR system. Gene‐specific forward and reverse primers were custom‐designed and synthesized by Macrogen (Republic of Korea). Primer sequences are listed in Table S1. Amplification reactions were carried out according to the manufacturer's recommended cycling conditions. Melt curve analysis was performed to confirm amplification specificity. Relative gene expression levels were normalized to GAPDH and calculated using the comparative 2^−^ΔΔCT method. Data are presented as fold change relative to the control group.

### 5.15 mRNA Sequencing

Total RNA was isolated from day 90 organoids using TRIzol Reagent (Invitrogen) according to the manufacturer's instructions. RNA quality and concentration were assessed using a NanoDrop Lite spectrophotometer (Thermo Scientific). RNA samples were submitted to a commercial sequencing service (Ebiogen, Seoul, Republic of Korea) for library preparation, next‐generation sequencing, and primary bioinformatic analysis. Briefly, poly(A)‐enriched mRNA libraries were constructed and sequenced on an Illumina platform to generate paired‐end reads. Raw sequencing data were subjected to quality control, adaptor trimming, and alignment to the reference genome using a standard RNA‐sequencing analysis pipeline. Gene expression levels were normalized to enable comparison between U‐hIEOs and M‐hIEOs. Global transcriptomic profiles, pathway enrichment analyses, and curated gene expression analyses were subsequently performed. Representative genes included in the mechanotransduction‐ and inner ear development‐associated heatmaps were selected based on established biological relevance together with expression differences between experimental groups. Functional enrichment analyses were performed using KEGG and Reactome databases.

### 5.16 Microfluidic Chip Fabrication

We designed our prototype using SolidWorks (Dassault Systèmes), a solid modeling computer‐aided design (CAD) software. The device was then fabricated using a commercially available high‐resolution DLP 3D printer. The resin used was MED‐AMB 10 (3D Systems), a rigid bio‐compatible resin capable of meeting ISO 10993‐5 and ‐10 standards for biocompatibility, cytotoxicity, sensitization, and irritation. The fabricated device was rinsed with isopropanol for 10 min and cured in a UV chamber for 1 h. Single‐sided PSA (Pressure Sensitive Adhesive) film (IS‐00820, IS Solution, Korea) was bound to the bottom of the device as PSA film is biocompatible and transparent similar to glass. After prototyping with the 3D printed platforms, the polystyrene (PS) microfluidic chip was fabricated through injection molding at an R&D Factory (South Korea). The aluminum alloy mold core was processed by machining and polishing. At the time of injection, the clamping force was set at 130 tons with a maximum injection pressure of 55 bar, 15 s of cycle time, and a 220°C nozzle temperature. The PSA film was bound to the injection‐molded PS part to complete the device.

### 5.17 Vascularization of M‐IEO

Human umbilical endothelial cells (HUVECs; Lonza; #C2519A) were subcultured into P4‐5 to be used for the experiment in endothelial growth medium 2 (EGM‐2; Lonza; #CC‐3162). Human brain vascular pericytes (HBVP; Sciencell; #1200) were cultured on 1 mg/ml poly‐L‐lysine (PLL, Sigma‐Aldrich) coated flasks and maintained in pericyte medium (Sciencell; #1201) and the cells between passages 3 and 4 were used for all experiments. For the hearing loss‐associated inflammation model and its treatments, recombinant human TNF‐α (R&D systems, USA) was diluted to 10 ng/ml with endothelial cell medium and applied to the media reservoirs. Infliximab (Selleckchem, USA) was diluted to 20 ng/ml with the endothelial cell medium and applied to the media reservoirs. The concentration was determined by referring to the reported in vitro inhibitory concentration of infliximab.

### 5.18 BLB Culture in a Microfluidic Chip

Before filling the microchannels, the 3D printed chips were irradiated with oxygen plasma using a plasma treatment system (Femto Science, Korea) at 70 W for 1 min to convert the hydrophobic surface to a hydrophilic one. A 50 µL suspension with 8 × 10^6^ cells/ml of HUVECs and HBVPs (HUVECs:HBVPs = 4:1, volume ratio), bovine fibrinogen solution (Sigma, USA; 2.5 mg/ml), and laminin from human placenta (Sigma, USA; 0.1 mg/ml) with 1 µL of bovine thrombin (Sigma, USA; 1 U/ml) was prepared. A 7 µL volume of this mixture was introduced into each central microchannel by injecting it through the central hole. The prepared EGM‐2 supplemented with vascular endothelial growth factor (VEGF; Peprotech; 50 ng/ml), fibroblast growth factor (FGF; Gibco; 20 ng/ml), and epidermal growth factor (EGF; Peprotech; 20 ng/ml) was applied to the media reservoir, after allowing 10 min for the patterned mixture to cross‐link at room temperature. The media supplemented with the growth factors was changed daily. After five days of culturing the HUVECs and HBVPs in an incubator at 37°C in 5% CO2, 25 days‐cultured IEOs were loaded on the vascular bed through the central hole of the microfluidic chip. After loading IEO, the IEO‐embedded vasculature was cultured for four days in an incubator at 37°C in 5% CO2.

### 5.19 Statistics and Reproducibility

All quantitative data are presented as mean ± s.e.m. Statistical analyses were performed using SigmaPlot 15.0 (Systat Software, Inc.). For comparisons between two groups, a two‐tailed unpaired Student's t‐test was used. For comparisons involving more than two groups, one‐way analysis of variance (ANOVA) followed by appropriate post hoc multiple comparison tests (Tukey's test) was applied, as specified in the corresponding figure legends. Correlation analyses were performed using Pearson's correlation coefficient with linear regression. Differences in variance between groups were assessed using an F‐test where appropriate. The exact statistical test used for each dataset is explicitly indicated in the corresponding figure legends. Biological replicates (n) refer to independent experiments performed using separate cell cultures or organoid batches, while technical replicates represent repeated measurements within the same experiment. The exact number of biological replicates for each experiment is provided in the respective figure legends. All experiments were performed with at least three independent biological replicates unless otherwise stated. Where applicable, individual data points are displayed in the graphs to represent biological or technical replicates. Representative images were selected from experiments showing consistent results across independent replicates. A *p*‐value < 0.05 was considered statistically significant.

## Funding

This work was supported by the National Research Foundation of Korea (NRF) grant funded by the Korean government (NRF‐2022M3A9H1015934, RS‐2024‐00337706, RS‐2022‐NR067329, RS‐2020‐NR049568, RS‐2025‐02216206, RS‐2025‐02243019, NRF‐2021R1A3B1077481). This work was also supported by the Korea Institute of Planning and Evaluation for Technology in Food, Agriculture and Forestry (IPET) through the Agriculture and Food Convergence Technologies Program for Research Manpower Development, funded by the Ministry of Agriculture, Food and Rural Affairs (MAFRA) (Project No. RS‐2024‐00397026). This work was supported by Korea Institute of Planning and Evaluation for Technology in Food, Agriculture and Forestry (IPET) through Companion Animal Intractable Disease Overcoming R&D Project, funded by Ministry of Agriculture, Food and Rural Affairs (MAFRA) (RS‐2026‐25519399). This research was also supported by the Commercialization Promotion Agency for R&D Outcomes (COMPA) funded by the Ministry of Science and ICT(MSIT) (RS‐2025‐25430866). This work was also supported by Korean Fund for Regenerative Medicine (KFRM) grant funded by the Korea government (the Ministry of Science and ICT, the Ministry of Health & Welfare) (22A0303L1‐01).

## Conflicts of Interest

The authors declare no conflicts of interest.

## Supporting information




**Supporting File**: adma74560‐sup‐0001‐SuppMat.docx.

## Data Availability

The authors confirm that the data supporting the findings of this study are available within the article and its supplementary information. Raw data that support the findings of this study are available from the corresponding author, upon reasonable request.

## References

[1] W. H. van der Valk , M. R. Steinhart , J. Zhang , and K. R. Koehler , “Building Inner Ears: Recent Advances and Future Challenges for in Vitro Organoid Systems,” Cell Death & Differentiation 28, no. 1 (2021): 24–34. 10.1038/s41418-020-00678-8 PMC7853146 33318601

[2] K. Connolly and A. Gonzalez‐Cordero , “Modelling Inner Ear Development and Disease Using Pluripotent Stem Cells—a Pathway to New Therapeutic Strategies,” Disease Models & Mechanisms 15, no. 11 (2022): dmm049593, 10.1242/dmm.049593. PMC10621662 36331565

[3] P.‐C. Tang , E. Hashino , and R. F. Nelson , “Progress in Modeling and Targeting Inner Ear Disorders With Pluripotent Stem Cells,” Stem Cell Reports 14, no. 6 (2020): 996–1008, 10.1016/j.stemcr.2020.04.008. PMC7355141 32442531

[4] D. Doda , S. Alonso Jimenez , H. Rehrauer , et al., “Human Pluripotent Stem Cell‐Derived Inner Ear Organoids Recapitulate Otic Development in Vitro,” Development 150, no. 19 (2023): dev201865, 10.1242/dev.201865. PMC10565253 37791525

[5] J. Qi , L. Zhang , X. Wang , et al., “Modeling, Applications and Challenges of Inner Ear Organoid,” Smart Medicine 3, no. 1 (2024): e20230028, 10.1002/SMMD.20230028. PMC11235738 39188517

[6] M. Hofer and M. P. Lutolf , “Engineering Organoids,” Nature Reviews Materials 6 (2021): 402–420, 10.1038/s41578-021-00279-y. PMC7893133 33623712

[7] X. Wu , F. Wang , R. Li , et al., “Enzyme‐Programmable DNA‐PEG Hydrogel Spatiotemporally Regulates Bone Regeneration Microenvironment,” Advanced Materials 38, no. 6 (2026): 14461, 10.1002/adma.202514461. 41178145

[8] R. Quan , X. Hou , J. Zhang , et al., “Spatiotemporally Engineered Protein Delivery via Integrated Covalent Coupling and Pathological Triggers for Precision Microenvironment Reprogramming in Tissue Injury Repair,” Advanced Materials 38, no. 1 (2026): 12554, 10.1002/adma.202512554. 40981608

[9] E. H. Finn and T. Misteli , “Molecular Basis and Biological Function of Variability in Spatial Genome Organization,” Science 365, no. 6457 (2019): aaw9498, 10.1126/science.aaw9498. PMC7421438 31488662

[10] S. A. Yi , Y. Zhang , C. Rathnam , T. Pongkulapa , and K.‐B. Lee , “Bioengineering Approaches for the Advanced Organoid Research,” Advanced Materials 33, no. 45 (2021): 2007949, 10.1002/adma.202007949. PMC8682947 34561899

[11] Z. Meng , K. Han , X. Huang , et al., “Micro/Nanoscale 3D Bioprinting: A Transformative Way to Modulate Cell/Tissue Growth and Function,” Advanced Materials 38 (2026): 15400. 10.1002/adma.202515400 41735735

[12] R. Z. Zhuang , F. Chesnais , and G. Vunjak‐Novakovic , “Integrating Hierarchical Vasculature Into Engineered Cardiac Tissues,” Advanced Materials 37, no. 47 (2025): 09242, 10.1002/adma.202509242. 41144916

[13] A. Puiggalí‐Jou , I. B. Hui , C. Fernandez‐Rico , and M. Zenobi‐Wong , “The Space Within: How Architected Voids Promote Tissue Formation,” Advanced Materials 38, no. 7 (2026): 07385. 10.1002/adma.202507385 41312612

[14] M. Nikkhah , F. Edalat , S. Manoucheri , and A. Khademhosseini , “Engineering Microscale Topographies to Control the Cell–Substrate Interface,” Biomaterials 33, no. 21 (2012): 5230–5246, 10.1016/j.biomaterials.2012.03.079. PMC3619386 22521491

[15] J. Kim , H. N. Kim , K.‐T. Lim , et al., “Designing Nanotopographical Density of Extracellular Matrix for Controlled Morphology and Function of human Mesenchymal Stem Cells,” Scientific Reports 3, no. 1 (2013): 3552, 10.1038/srep03552. PMC6506445 24352057

[16] J. Kim , W.‐G. Bae , Y. J. Kim , et al., “Directional Matrix Nanotopography With Varied Sizes for Engineering Wound Healing,” Advanced Healthcare Materials 6, no. 19 (2017): 1700297, 10.1002/adhm.201700297. 28636203

[17] W. Kim , Y. Gwon , Y. K. Kim , et al., “Plasma‐Assisted Multiscale Topographic Scaffolds for Soft and Hard Tissue Regeneration,” npj Regenerative Medicine 6, no. 1 (2021): 52. 10.1038/s41536-021-00162-y PMC8429553 34504097

[18] N. Gjorevski , M. Nikolaev , T. E. Brown , et al., “Tissue Geometry Drives Deterministic Organoid Patterning,” Science 375, no. 6576 (2022): aaw9021, 10.1126/science.aaw9021. PMC9131435 34990240

[19] K. Ishihara , et al., “Topological Morphogenesis of Neuroepithelial Organoids,” Nature Physics 19, no. 2 (2023): 177–183. 10.1038/s41567-022-01822-6 PMC9928582 36815964

[20] G. Rossi , A. Manfrin , and M. P. Lutolf , “Progress and Potential in Organoid Research,” Nature Reviews Genetics 19, no. 11 (2018): 671–687, 10.1038/s41576-018-0051-9. 30228295

[21] P. C. Uzabakiriho , F. Jiajun , B. A. Nguchu , S. Iqbal , C. Manishimwe , and P. Shaw , “Spheroid‐on‐a‐Chip Platforms for Tumor Microenvironment and Drug Development,” Advanced Materials Technologies 10, no. 11 (2025): 2401821, 10.1002/admt.202401821.

[22] S. Bhakuni , H. Sharma , W. Kim , et al., “Micro and Nanoengineered Cardiac Spheroids and Organoids: Toward Translational Applications,” Advanced Functional Materials 36, no. 33 (2026): 31032, 10.1002/adfm.202531032.

[23] N. Brandenberg , S. Hoehnel , F. Kuttler , et al., “High‐throughput Automated Organoid Culture via Stem‐Cell Aggregation in Microcavity Arrays,” Nature Biomedical Engineering 4, no. 9 (2020): 863–874, 10.1038/s41551-020-0565-2. 32514094

[24] G. H. Lee , J. S. Lee , G.‐H. Lee , et al., “Networked Concave Microwell Arrays for Constructing 3D Cell Spheroids,” Biofabrication 10, no. 1 (2018): 015001, 10.1088/1758-5090/aa9876. 29190216

[25] Z. Chen and R. Zhao , “Engineered Tissue Development in Biofabricated 3D Geometrical Confinement–A Review,” ACS Biomaterials Science & Engineering 5, no. 8 (2019): 3688–3702, 10.1021/acsbiomaterials.8b01195. 33405885

[26] C.‐K. Huang , G. J. Paylaga , S. Bupphathong , and K.‐H. Lin , “Spherical Microwell Arrays for Studying Single Cells and Microtissues in 3D Confinement,” Biofabrication 12, no. 2 (2020): 025016, 10.1088/1758-5090/ab6eda. 31974317

[27] K. R. Koehler and E. Hashino , “3D Mouse Embryonic Stem Cell Culture for Generating Inner Ear Organoids,” Nature Protocols 9, no. 6 (2014): 1229–1244, 10.1038/nprot.2014.100. PMC6453571 24784820

[28] R. E. DeJonge , X.‐P. Liu , C. R. Deig , S. Heller , K. R. Koehler , and E. Hashino , “Modulation of Wnt Signaling Enhances Inner Ear Organoid Development in 3D Culture,” PLoS ONE 11, no. 9 (2016): 0162508, 10.1371/journal.pone.0162508. PMC5015985 27607106

[29] T. Self , M. Mahony , J. Fleming , J. Walsh , S. D. M. Brown , and K. P. Steel , “Shaker‐1 Mutations Reveal Roles for Myosin VIIA in both Development and Function of Cochlear Hair Cells,” Development 125, no. 4 (1998): 557–566, 10.1242/dev.125.4.557. 9435277

[30] C. R. Hume , D. L. Bratt , and E. C. Oesterle , “Expression of LHX3 and SOX2 During Mouse Inner Ear Development,” Gene Expression Patterns 7, no. 7 (2007): 798–807, 10.1016/j.modgep.2007.05.002. PMC2043117 17604700

[31] M. C. Liberman , J. Gao , D. Z. Z. He , X. Wu , S. Jia , and J. Zuo , “Prestin Is Required for Electromotility of the Outer Hair Cell and for the Cochlear Amplifier,” Nature 419, no. 6904 (2002): 300–304, 10.1038/nature01059. 12239568

[32] R. P. Seal , O. Akil , E. Yi , et al., “Sensorineural Deafness and Seizures in Mice Lacking Vesicular Glutamate Transporter 3,” Neuron 57, no. 2 (2008): 263–275, 10.1016/j.neuron.2007.11.032. PMC2293283 18215623

[33] J. E. Gale , W. Marcotti , H. J. Kennedy , C. J. Kros , and G. P. Richardson , “FM1‐43 Dye Behaves as a Permeant Blocker of the Hair‐Cell Mechanotransducer Channel,” The Journal of Neuroscience 21, no. 18 (2001): 7013–7025, 10.1523/JNEUROSCI.21-18-07013.2001. PMC6762973 11549711

[34] M. Roccio , M. Perny , M. Ealy , H. R. Widmer , S. Heller , and P. Senn , “Molecular Characterization and Prospective Isolation of human Fetal Cochlear Hair Cell Progenitors,” Nature Communications 9, no. 1 (2018): 4027, 10.1038/s41467-018-06334-7. PMC6168603 30279445

[35] X.‐P. Liu , K. R. Koehler , A. M. Mikosz , E. Hashino , and J. R. Holt , “Functional Development of Mechanosensitive Hair Cells in Stem Cell‐Derived Organoids Parallels Native Vestibular Hair Cells,” Nature Communications 7, no. 1 (2016): 11508, 10.1038/ncomms11508. PMC4890183 27215798

[36] T. Karasawa and P. S. Steyger , “An Integrated View of Cisplatin‐Induced Nephrotoxicity and Ototoxicity,” Toxicology Letters 237, no. 3 (2015): 219–227, 10.1016/j.toxlet.2015.06.012. PMC4516600 26101797

[37] M. Jeong , M. O'Reilly , N. K. Kirkwood , et al., “Generating Inner Ear Organoids Containing Putative Cochlear Hair Cells From human Pluripotent Stem Cells,” Cell Death & Disease 9, no. 9 (2018): 922, 10.1038/s41419-018-0967-1. PMC6134051 30206231

[38] M. R. Steinhart , W. H. Van der Valk , and D. Osorioet , “Mapping Oto‐Pharyngeal Development in a Human Inner Ear Organoid Model,” Development 150, no. 19 (2023): dev201871. 10.1242/dev.201871 PMC10698753 37796037

[39] A. M. Binshtok , P. Gerner , S. B. Oh , et al., “Coapplication of Lidocaine and the Permanently Charged Sodium Channel Blocker QX‐314 Produces a Long‐Lasting Nociceptive Blockade in Rodents,” Anesthesiology 111, no. 1 (2009): 127–137, 10.1097/ALN.0b013e3181a915e7. PMC2761747 19512868

[40] G. Halder , S. Dupont , and S. Piccolo , “Transduction of Mechanical and Cytoskeletal Cues by YAP and TAZ,” Nature Reviews Molecular Cell Biology 13, no. 9 (2012): 591–600, 10.1038/nrm3416. 22895435

[41] A. Totaro , T. Panciera , and S. Piccolo , “YAP/TAZ Upstream Signals and Downstream Responses,” Nature cell biology 20 (2018): 888–899. 10.1038/s41556-018-0142-z PMC6186418 30050119

[42] A. E. Kiernan , “Notch Signaling During Cell Fate Determination in the Inner Ear,” Seminars in Cell & Developmental Biology 24, no. 5 (2013): 470–479, 10.1016/j.semcdb.2013.04.002. PMC3725958 23578865

[43] R. Cohen and D. Sprinzak , “Mechanical Forces Shaping the Development of the Inner Ear,” Biophysical Journal 120 (2021): 4142–4148. 10.1016/j.bpj.2021.06.036 PMC8516635 34242589

[44] S. Dupont , “Role of YAP/TAZ in Cell‐Matrix Adhesion‐Mediated Signalling and Mechanotransduction,” Experimental cell research 343 (2422): 42–53. 10.1016/j.yexcr.2015.10.034 26524510

[45] S. T. Moore , T. Nakamura , J. Nie , et al., “Generating High‐fidelity Cochlear Organoids From human Pluripotent Stem Cells,” Cell Stem Cell 30, no. 7 (2023): e950–961.e7, 10.1016/j.stem.2023.06.006. PMC10695300 37419105

[46] A. Armulik , G. Genové , M. Mäe , et al., “Pericytes Regulate the Blood–Brain Barrier,” Nature 468, no. 7323 (2010): 557–561, 10.1038/nature09522. 20944627

[47] M. G. Andrews and A. R. Kriegstein , “Challenges of Organoid Research,” Annual Review of Neuroscience 45 (2022): 23–39, 10.1146/annurev-neuro-111020-090812. PMC10559943 34985918

[48] S. Goli , A. T. Guduru , S. Karadagatla , et al., “Generation of Uniform‐Sized Spheroids as 3D Cancer Models Using Simple and Scalable PDMS‐Based Microwell Devices,” RSC Advances 16, no. 6 (2026): 5680–5691, 10.1039/D5RA07878D. PMC12834093 41602196

[49] A. Warmflash , B. Sorre , F. Etoc , E. D. Siggia , and A. H. Brivanlou , “A Method to Recapitulate Early Embryonic Spatial Patterning in Human Embryonic Stem Cells,” Nature Methods 11, no. 8 (2014): 847–854, 10.1038/nmeth.3016. PMC4341966 24973948

[50] W. Bi , S. Cai , C. Xing , L. Wang , and H. Du , “Engineering Mesenchymal Stem Cell Spheroids and Brain Organoids: Advanced 3D Culture Platforms for Neurodegenerative Disease Cell Therapy,” Stem Cell Reviews and Reports 22, no. 4 (2026): 1739–1763, 10.1007/s12015-026-11101-z. 41874927

[51] V. Velasco , S. A. Shariati , and R. Esfandyarpour , “Microtechnology‐based Methods for Organoid Models,” Microsystems & Nanoengineering 6, no. 1 (2020): 76, 10.1038/s41378-020-00185-3. PMC8433138 34567686

[52] D. S. André and C. Labouesse , “Mechanobiology of 3D Cell Confinement and Extracellular Crowding,” Biophysical Reviews 16, no. 6 (2024): 833–849. 10.1007/s12551-024-01244-z PMC11735831 39830117

[53] S. Grebenyuk and A. Ranga , “Engineering Organoid Vascularization,” Frontiers in Bioengineering and Biotechnology 7 (2019): 39, 10.3389/fbioe.2019.00039. PMC6433749 30941347

[54] S. Zhang , Z. Wan , and R. D. Kamm , “Vascularized Organoids on a Chip: Strategies for Engineering Organoids With Functional Vasculature,” Lab on a Chip 21, no. 3 (2021): 473–488, 10.1039/D0LC01186J. PMC8283929 33480945

[55] J. D. Humphrey , E. R. Dufresne , and M. A. Schwartz , “Mechanotransduction and Extracellular Matrix Homeostasis,” Nature reviews Molecular cell biology 15 (2014): 802–812. 10.1038/nrm3896 PMC4513363 25355505

[56] S. Dupont , L. Morsut , M. Aragona , et al., “Role of YAP/TAZ in Mechanotransduction,” Nature 474 (2011): 179–183. 10.1038/nature10137 21654799

[57] B. Geiger , A. D. Spatz Jp Fau‐Bershadsky , and A. D. Bershadsky , “Environmental Sensing Through Focal Adhesions,” Nature reviews Molecular cell biology 10 (2009): 21–33. 10.1038/nrm2593 19197329

[58] R. Nusse and H. Clevers , “Wnt/β‐Catenin Signaling, Disease, and Emerging Therapeutic Modalities,” Cell 169 (2017): 985–999. 10.1016/j.cell.2017.05.016 28575679

[59] V. Munnamalai and D. M. Fekete , “Wnt Signaling During Cochlear Development,” Seminars in Cell & Developmental Biology 24, no. 5 (2013): 480–489, 10.1016/j.semcdb.2013.03.008. PMC3690158 23548730

[60] R. Cohen , L. Amir‐Zilberstein , M. Hersch , et al., “Mechanical Forces Drive Ordered Patterning of Hair Cells in the Mammalian Inner Ear,” Nature Communications 11, no. 1 (2020): 5137, 10.1038/s41467-020-18894-8. PMC7550578 33046691

[61] L. J. Chacko , C. Sergi , T. Eberharter , et al., “Early Appearance of Key Transcription Factors Influence the Spatiotemporal Development of the human Inner Ear,” Cell and Tissue Research 379, no. 3 (2020): 459–471, 10.1007/s00441-019-03115-6. 31788757

[62] K. H. Vining and D. J. Mooney , “Mechanical Forces Direct Stem Cell Behaviour in Development and Regeneration,” Nature Reviews Molecular Cell Biology 18, no. 12 (2017): 728–742, 10.1038/nrm.2017.108. PMC5803560 29115301

[63] K. R. Koehler , A. M. Mikosz , A. I. Molosh , D. Patel , and E. Hashino , “Generation of Inner Ear Sensory Epithelia From Pluripotent Stem Cells in 3D Culture,” Nature 500, no. 7461 (2013): 217–221, 10.1038/nature12298. PMC3739998 23842490

[64] J. R. Meyers , R. B. MacDonald , A. Duggan , et al., “Lighting up the Senses: FM1‐43 Loading of Sensory Cells Through Nonselective Ion Channels,” The Journal of Neuroscience 23, no. 10 (2003): 4054–4065, 10.1523/JNEUROSCI.23-10-04054.2003. PMC6741082 12764092

[65] K. Oshima , K. Shin , M. Diensthuber , A. W. Peng , A. J. Ricci , and S. Heller , “Mechanosensitive Hair Cell‐Like Cells From Embryonic and Induced Pluripotent Stem Cells,” Cell 141, no. 4 (2010): 704–716, 10.1016/j.cell.2010.03.035. PMC2873974 20478259

[66] P. G. Gillespie and U. Müller , “Mechanotransduction by Hair Cells: Models, Molecules, and Mechanisms,” Cell 139, no. 1 (2009): 33–44, 10.1016/j.cell.2009.09.010. PMC2888516 19804752

[67] I. Omana‐Zapata , M. A. Khabbaz , J. C. Hunter , and K. R. Bley , “QX‐314 Inhibits Ectopic Nerve Activity Associated With Neuropathic Pain,” Brain Research 771, no. 2 (1997): 228–237, 10.1016/S0006-8993(97)00770-1. 9401743

[68] X. Shi , “Pathophysiology of the Cochlear Intrastrial Fluid‐blood Barrier (review),” Hearing Research 338 (2016): 52–63, 10.1016/j.heares.2016.01.010. PMC5322264 26802581

[69] L. Neng and X. Shi , “Vascular Pathology and Hearing Disorders,” Current Opinion in Physiology 18 (2020): 79–84, 10.1016/j.cophys.2020.09.004.

[70] M. Sekulic , N. Abdollahi , L. Graf , et al., “Human Blood‐Labyrinth Barrier on a Chip: A Unique in Vitro Tool for Investigation of BLB Properties,” RSC Advances 13, no. 36 (2023): 25508–25517, 10.1039/D3RA04704K. PMC10450574 37636514

[71] E. M. Keithley , X. Wang , and G. C. Barkdull , “Tumor Necrosis Factor α Can Induce Recruitment of Inflammatory Cells to the Cochlea,” Otology & Neurotology 29, no. 6 (2008): 854–859, 10.1097/MAO.0b013e31818256a9. 18636025

[72] S. K. Moon , J.‐I. Woo , and D. J. Lim , “Involvement of TNF‐α and IFN‐γ in Inflammation‐Mediated Cochlear Injury,” Annals of Otology, Rhinology & Laryngology 128, no. 6q (2019): 8S–15S, 10.1177/0003489419837689. 31092034

[73] Z. Wen , M. Orduno , Z. Liang , X. Gong , and M. Mak , “Optimization of Vascularized Intestinal Organoid Model,” Advanced Healthcare Materials 13, no. 31 (2024): 2400977, 10.1002/adhm.202400977. PMC11652258 39091070

[74] V. L. Silvestri , E. Henriet , R. M. Linville , A. D. Wong , P. C. Searson , and A. J. Ewald , “A Tissue‐Engineered 3D Microvessel Model Reveals the Dynamics of Mosaic Vessel Formation in Breast Cancer,” Cancer Research 80, no. 19 (2020): 4288–4301, 10.1158/0008-5472.CAN-19-1564. PMC7541732 32665356

[75] B. Palikuqi , D.‐H. T. Nguyen , G. Li , et al., “Adaptable Haemodynamic Endothelial Cells for Organogenesis and Tumorigenesis,” Nature 585, no. 7825 (2020): 426–432, 10.1038/s41586-020-2712-z. PMC7480005 32908310

