# Supplementary material for: Engineering Pluripotent Stem Cells‐Derived Inner Ear Organoids With Enhanced Maturation and Reproducibility by Micro‐Topographical Cues
Source: Adv Mater. 2026 Aug 10;38(57):e74560. doi: 10.1002/adma.74560 (PMC13660830; doi:10.1002/adma.74560)
Supplement: Supplementary file 1 [file ADMA-38-e74560-s001.docx]

Supporting Information

**Engineering Pluripotent Stem Cells-derived Inner Ear Organoids with Enhanced Maturation and Reproducibility by Micro-Topographical Cues**

*Harshita Sharma^+^, Jungeun Lim^+^, Woochan Kim^+^, Yeon Ju Kim^+^, Dream Kim, Shinyull Lee, Chaeyeon Park, Stephen Rhee, Shruthy Kuttapan, Jungho Ha, Sang Wook Park, Kyunghoon Kim, Noo Li Jeon, Sunho Park*, Yun-Hoon Choung*, Jangho Kim**

**Figure S1. Optimization of initial cell seeding density for spheroid formation. a-b)** Bright-field images showing spheroid formation at different initial cell seeding densities (MS: 2 × 10³, MM: 3 × 10³, ML-Sph: 4 × 10³ cells per well) at early time points. **c-e)** Morphological progression and compaction of spheroids over time demonstrate increased structural integrity at higher seeding densities. **f-g)** Immunofluorescence staining reveals cytoskeletal organization and epithelial characteristics, with phalloidin labeling F-actin and integrin β4 (ITGB4) indicating enhanced epithelial features in ML-Sph compared to lower-density conditions. Scale bars, as indicated. Plots show mean ± s.e.m. from n ≥ 3 independent biological replicates (individual data points represent technical replicates). The statistical test used is indicated for each comparison. *p < 0.05, **p < 0.01, ***p < 0.001.

**Figure S2. Structural maturation and basement membrane-like organization following Matrigel supplementation. a-c)** Morphological and ultrastructural analysis of ML-Sph aggregates following Matrigel supplementation, showing the development of a dense outer layer. **d-e)** Scanning electron microscopy (SEM) and surface topology analysis confirm the presence of a compact external structure consistent with basement membrane-like organization. **f)** Immunofluorescence staining shows increased laminin β1 (LAMB1) expression and reduced OCT4 levels, indicating progression from pluripotency toward early lineage commitment. **g-h)** Expression of CX43 and CyclinD1 suggests intercellular connectivity and proliferative capacity during early stages. Scale bars, as indicated. Plots show mean ± s.e.m. from n ≥ 3 independent biological replicates (individual data points represent technical replicates). The statistical test used is indicated for each comparison. *p < 0.05, **p < 0.01, ***p < 0.001.

**Figure S3. Comparison of structural integrity and epithelial organization across seeding densities. a-b)** Bright-field and morphological analysis showing structural differences between lower-density (MS, MM) and higher-density (ML-Sph) aggregates, with lower-density conditions exhibiting irregular morphology and reduced surface integrity. **c)** Immunofluorescence staining at day 14 for the otic markers PAX8 (red), E-CAD (green), and DAPI (blue). White dashed boxes indicate regions of localized epithelial organization. Scale bars, 100 µm. **d)** High-magnification bright-field image of a day 10 ML-Sph aggregate showing early surface protrusions. Scale bar, 50 µm. **e)** Quantification of mean fluorescence intensity for PAX8 and E-CAD across the different groups. **f)** High-magnification views of the regions highlighted in (c), demonstrating the formation of distinct otic vesicle-like structures in ML-Sph compared to the more disorganized morphology in U-Sph. Scale bars, 50 µm. Plots show mean ± s.e.m. from n ≥ 3 independent biological replicates (individual data points represent technical replicates). The statistical test used is indicated for each comparison. *p < 0.05, **p < 0.01, ***p < 0.001.

**Figure S4. Comparative structural analysis and cisplatin-induced ototoxicity in M-mIEOs vs. U-mIEOs. a-b)** Scanning electron microscopy (SEM) images of (a) U-mIEO and (b) M-mIEO at day 30, showing the outer surface morphology (left), whole organoid view (center), and high-magnification views of the internal torn surface (right). Scale bars as indicated. **c** Quantification of the aggregate formation number per 100 wells for U- and M-conditions. **d)** Average number of budding structures and vesicles per organoid. **e)** Morphological comparison of organoid perimeter and total area. **f)** Distribution of individual organoid areas for U-mIEO and M-mIEO, with horizontal lines representing the mean values. **g)** Representative immunofluorescence images of U-mIEO and M-mIEO following cisplatin (+Cis) treatment, stained for the hair cell marker MYO7A (green), progenitor marker SOX2 (red), and DAPI (blue). Scale bars, 20 µm. **h)** Quantification of MYO7A+ and SOX2+ cell populations expressed as a percentage of total cells following cisplatin treatment. **i)** Quantitative RT-PCR analysis of gene expression related to hair cells (Myo7a, Espin, Pouf3), neurons (Tubb3, Neun, Nestin), and synapses (Syn1, Slc17a8, Ctbp2) in U-mIEO and M-mIEO under control and cisplatin-treated conditions. Plots show mean ± s.e.m. from n ≥ 3 independent biological replicates (individual data points represent technical replicates). The statistical test used is indicated for each comparison. *p < 0.05, **p < 0.01, ***p < 0.001.

**Figure S5. Comparative transcriptomic profiling and pathway enrichment analysis of M-hIEOs. a-b)** Box plots showing the distribution of mRNA expression levels for grouped up- and down-regulated genes (a) and genes classified by developmental lineage and cellular compartment (b). **c)** Comparative expression analysis of cochlear- and vestibular-associated genes in organoids showing relative mRNA expression (log2 fold change) of selected cochlear hair cell-associated markers (left panel) and vestibular hair cell-associated markers (right panel) in microengineered organoids. Specific categories include surface ectoderm, extracellular matrix (ECM), otic vesicles, neurosensory cells, sensory epithelium, supporting cells, and hair cells. M-hIEOs exhibit a consistent upward shift in expression across mature otic lineages. **d)** Reactome pathway enrichment analysis showing significantly enriched biological processes in M-hIEOs (red) versus U-hIEOs (blue). Key enriched clusters include cell cycle regulation, cell differentiation, and angiogenesis-related signaling, supporting the enhanced maturation and vascular-receptive state of the microengineered organoids. Plots show mean ± s.e.m. from n ≥ 3 independent biological replicates. The statistical test used is indicated for each comparison. *p < 0.05, **p < 0.01, ***p < 0.001.

**Figure S6. Enhanced developmental and lineage-specific marker expression in M-hIEOs. a-g)** Quantitative RT-qPCR analysis of markers associated with distinct developmental stages and cellular lineages in U-hIEO (blue) and M-hIEO (red) platforms. Gene expression is normalized to GAPDH and shown relative to the U-hIEO control. **h)** Schematic illustration highlighting the role of topographical cues provided by the microwell chip in orchestrating the accelerated transcriptomic maturation and synchronized gene expression patterns observed in M-hIEOs. Plots show mean ± s.e.m. from n ≥ 3 independent biological replicates (individual data points represent technical replicates). The statistical test used is indicated for each comparison. *p < 0.05, **p < 0.01, ***p < 0.001.

**Figure S7. Design and fluidic characterization of the microfluidic organoid-on-chip platform. a)** 3D CAD rendering of the microfluidic chip architecture. The central chamber (red inset) is designed for the precise positioning of the inner ear organoid (IEO) within an engineered extracellular matrix (ECM) scaffold. **b)** Schematic of the spontaneous capillary pinning mechanism at the corner geometries. The Concus-Pound condition governs the liquid-air interface stability, ensuring the hydrogel (blue) remains confined within the central chamber while exerting uniform surface tension-driven mechanical cues on the organoid (yellow). **c)** 3D CAD rendering highlighting the side channel architecture (blue inset). These channels are utilized for the delivery of nutrients, morphogens, and the establishment of the vascular network comprising endothelial cells (ECs) and pericytes (PCs). **d)** Schematic representation of the fluidic interface between the central organoid chamber and the adjacent side channels. The design enables stable co-culture and biochemical crosstalk through a controlled diffusion interface, facilitating the reconstitution of the Blood-Labyrinth Barrier (BLB)-mimetic environment without disruptive shear stress on the developing organoid.

**Table S1:** List of genes along with forward and reverse primer sequences.

| **Genes** | **Forward sequence** | **Reverse sequence** |
| --- | --- | --- |
| GADPH | 5’-AACGGGAAGCCCATCACC-3’ | 5’- CAGCCTTGGCAGCACCAG-3 |
| m-OCT4 | 5-ATGGCATACTGTGGACCTCA-3‘ | 5‘-AGCAGCTTGGCAAACTGTTC-3‘ |
| h-OCT4 | 5‘-CCTGAAGCAGAAGAGGATCACC-3‘ | 5‘-AAAGCGGCAGATGGTCGTTTGG-3‘ |
| m-LAMININ β1 | 5‘-GCTGCTCAATTATGCCAAGAAG-3‘ | 5‘-GCCGCATCCTTAGAGTTTAGT-3‘ |
| h-LAMININ β1 | 5‘-GAGGTGTCTCAAGTGCCTGTAC-3‘ | 5‘-ACTGGCAGTCAGAGCCGTTACA-3‘ |
| m-GATA2 | 5‘-CTTCAACCATCTCGACTCGCAG-3‘ | 5‘-GCAACAAGTGTGGTCGGCACAT-3‘ |
| h-GATA2 | 5‘-CAGCAAGGCTCGTTCCTGTTCA-3‘ | 5‘-ATGAGTGGTCGGTTCTGCCCAT-3‘ |
| m-SIX1 | 5‘-AGGTCAGCAACTGGTTTAAGAACC-3‘ | 5‘-GAGTTGATTCTGCTTGTTGGAGG-3‘ |
| m-PAX8 | 5‘-GCCAAGTGCTCTCCTGTGTC-3‘ | 5‘-CTTTGCAGTCCCCAGCTCAG-3‘ |
| h-PAX8 | 5‘-TCAACCTCCCTATGGACAGCTG-3‘ | 5‘-GAGCCCATTGATGGAGTAGGTG-3‘ |
| m-ECAD | 5‘-TCTTAGGCACCCAGTAGGCC-3‘ | 5‘-TTCCAGGGAGACTGCTAGGC-3‘ |
| h-ECAD | 5‘-GCCTCCTGAAAAGAGAGTGGAAG-3‘ | 5‘-TGGCAGTGTCTCTCCAAATCCG-3‘ |
| m-JAG1 | 5‘-TGCGTGGTCAATGGAGACTCCT-3‘ | 5‘-TCGCACCGATACCAGTTGTCTC-3‘ |
| m-ATOH1 | 5‘-ACATCTCCCAGATCCCACAG-3‘ | 5‘-ACAACGATCACCACAGACCA-3‘ |
| h-ATOH1 | 5‘-CCTTCCAGCAAACAGGTGAATGG-3‘ | 5‘-GAACGACGGGATAACATTGCGC-3‘ |
| m-MYO7A | 5’-TCCTGCAGTGCCACCACATA-3’ | 5’-TGGCCACCATCAAAGGAGAT-3’ |
| h-MYO7A | 5‘-CCCAAGAACGACGTCATC-3‘ | 5‘-GTTTTCGCTCCCCTGCT-3‘ |
| m-ESPIN | 5‘-GCAGAAGATGCAGGAGGAAG-3‘ | 5‘-GACTGTTCTTTCGCCCTCTG-3‘ |
| h-ESPIN | 5‘-TGCAGACCAAGAACAAACTCCGC-3‘ | 5‘-TTGCGGCTGGAGTCCTGCCTC-3‘ |
| m-PRESTIN | 5‘-CGACTTGTATAGCAGCGCTTTAAA-3‘ | 5‘-TTCTTCTCGCTCCCATAATGAGT-3‘ |
| h-PRESTIN | 5‘-AAGAGGCAGCGGCTGTG-3‘ | 5‘-TGAGGCTGAGAGCAGGAGAA-3‘ |
| m-DNM3 | 5‘-AACTTCACATCAACGCGACC-3‘ | 5‘-CTCGCACTGGAGTCTCTGAT-3‘ |
| h-DNM3 | 5‘-GCTCACCATCAGCAACATTGGC-3‘ | 5‘-CCGAACTTTCAGGTTGTCCAAGG-3‘ |
| m-OCM | 5‘-GACAACGACCAGAGTGGATACC-3‘ | 5‘-CCATCAAGGACTTGGTCTCTGAC-3‘ |
| h-OCM | 5‘-CGGTTCATAGACAACGACCAGAG-3‘ | 5‘-CATTATCCGCCGCAGCCATCAA-3‘ |
| m-NESTIN | 5‘-GCTTCTCTTGGCTTTCCTGA-3‘ | 5‘-AGAGAAGGATGTTGGGCTGA-3‘ |
| h-NESTIN | 5‘-TCAAGATGTCCCTCAGCCTGGA-3‘ | 5‘-AAGCTGAGGGAAGTCTTGGAGC-3‘ |
| m-TUJ1 | 5‘-TCACCACCTCCCTTCGATTC-3‘ | 5‘-CACCATGTTCACAGCCAGCTT-3‘ |
| h-TUJ1 | 5‘-TCAGCGTCTACTACAACGAGGC-3‘ | 5‘-GCCTGAAGAGATGTCCAAAGGC-3‘ |
| m-CAT | 5’-TGAGAAGCCTAAGAACGCAATTC-3’ | 5’-CCCTTCGCAGCCATGTG-3’ |
| m-SOD2 | 5’-TTA ACGCGCAGATCATGCA-3’ | 5’-GGTGGCGTTGAGATTGTTCA-3’ |
| m-GPx | 5‘-CCTCTGCTGCAAGAGCCTCCC | CTTATCCAGGCAGACCATGTGC |
| m-NRF2 | 5’-CGAGATATACGCAGGAGAGGTAAG A-3’ | 5’-GCTCGACAATGTTCTCCAGCTT-3’ |
| m-Stat1 | 5’-TCACAGTGGTTCGAGCTTCAG-3’ | 5’-GCAAACGAGACATCATAGGCA-3’ |
| m-IL-6 | 5’-CTCCGACTTGTGAAGTGGTAT AG-3’ | 5’-CTTCC TCCAGTTGCCTTCT-3’ |
| h-SLC2A3 | 5’-TGCCTTTGGCACTCTCAACCAG-3’ | 5’-GCCATAGCTCTTCAGACCCAAG-3’ |
| h-TFAP2A | 5’-GACCTCTCGATCCACTCCTTAC-3’ | 5’-GAGACGGCATTGCTGTTGGACT-3’ |
| h-CDH11 | 5’-GATCGTCACACTGACCTCGACA-3’ | 5’-CTTTGGCTTCCTGATGCCGATTG-3’ |
| h-PDGFRA | 5’-GACTTTCGCCAAAGTGGAGGAG-3’ | 5’-AGCCACCGTGAGTTCAGAACGC-3’ |
| h-TRPM3 | 5’-GGAAGTGTTTGCGGACCAGATAG-3’ | 5’-GCACGATCCAAGCTCCTGTCTT-3’ |
| h-EPCAM | 5’-GCCAGTGTACTTCAGTTGGTGC-3’ | 5’-CCCTTCAGGTTTTGCTCTTCTCC-3’ |
| h-DCC | 5’-GGTGAGATGGAAACACTGGAGC-3’ | 5’-CTGTCATGGCTGACACCTGGAA-3’ |
| h-PAX2 | 5’-GACTATGTTCGCCTGGGAGATTC-3’ | 5’-AAGGCTGCTGAACTTTGGTCCG-3’ |
| h-EPHA7 | 5’-CTCGCAAGTGAGCGGAGTAATG-3’ | 5’-GTTGAGTAGGTCCGTTCCCTTTG-3’ |
| h-SPP1 | 5’-CGAGGTGATAGTGTGGTTTATGG-3’ | 5’-GCACCATTCAACTCCTCGCTTTC-3’ |
| h-ZBTB20 | 5’-CTCTGCAACAAGACTTTCACCGC-3’ | 5’-AGGAGAAGGAGCGCCAACAGAT-3’ |
| h-LIN28B | 5’-CCTGTTTAGGAAGTGAAAGAAGAC-3’ | 5’-CACTTCTTTGGCTGAGGAGGTAG-3’ |
| h-STRC | 5’-CCTTTCTTGGCTGTCTCTGACC-3’ | 5’-TCGCTTTGCCAGAGCCTCCTTC-3’ |
| h-SMPX | 5’-TTTCGGCCAGGAGCAGGTCAAC-3’ | 5’-GTTTCTTCGCTCCTGGAATTGGC-3’ |
| m-Occludin | 5’-CGGCAATGAAACAAAAGGCAG-3’ | 5’-GGCTATGGTTATGGCTATGGCTAC-3’ |
| m-Claudin-1 | 5’-GTCTTTGACTCCTTGCTGAATCTG-3’ | 5’-CACCTCATCGTCTTCCAAGCAC-3 |
| m-ZO-1 | 5-CGGTCCTCTGAGCCTGTAAG-3 | 5-GGATCTACATGCGACGACAA-3 |

Table S2: Representative mechanotransduction-associated genes identified by RNA sequencing and used for transcriptomic analyses.

| **Category** | **Gene Symbol** | **Normalized expression** | | **Log2 Fold change** | |
| --- | --- | --- | --- | --- | --- |
|  |  | U-hIEO | M-hIEO | U-hIEO | M-hIEO |
| Focal Adhesion/Integrin Signalling | ITGA1 | 2.165931504 | 4.741095883 | -1.28758219 | 1.28758219 |
|  | ITGA5 | 4.44297753 | 6.022700143 | -0.789861307 | 0.789861307 |
|  | TLN2 | 5.64933674 | 6.789501123 | -0.570082191 | 0.570082191 |
|  | ZYX | 5.593781515 | 6.968109223 | -0.687163854 | 0.687163854 |
|  | RAP1GAP2 | 4.158111476 | 5.938101038 | -0.889994781 | 0.889994781 |
|  | AKT3 | 6.440388018 | 5.331023628 | 0.554682195 | -0.554682195 |
|  | PRKCA | 3.480858553 | 4.927628005 | -0.723384726 | 0.723384726 |
|  | HRAS | 4.284359836 | 3.165187106 | 0.559586365 | -0.559586365 |
|  | MAP2K3 | 3.367648349 | 4.485496686 | -0.558924168 | 0.558924168 |
| Actin Cytoskeleton | ACTA2 | 3.641708596 | 5.132574408 | -0.745432906 | 0.745432906 |
|  | MYL9 | 4.635628074 | 6.249079233 | -0.80672558 | 0.80672558 |
|  | FHOD1 | 3.337891725 | 4.959990657 | -0.811049466 | 0.811049466 |
|  | DIAPH2 | 4.341691014 | 5.678292331 | -0.668300659 | 0.668300659 |
|  | ACTA1 | 1.526584055 | 4.73455011 | -1.603983027 | 1.603983027 |
|  | MYL1 | 0.507241772 | 5.272615509 | -2.382686868 | 2.382686868 |
|  | TWNK | 3.683680695 | 4.716559022 | -0.516439163 | 0.516439163 |
|  | MAP7D3 | 4.364001895 | 5.644300111 | -0.640149108 | 0.640149108 |
|  | MYH9 | 9.898476853 | 9.179024636 | 0.359726109 | -0.359726109 |
| Hippo Signaling | WWTR1 (TAZ) | 5.083912121 | 6.240722222 | -0.57840505 | 0.57840505 |
|  | TEAD4 | 3.672890589 | 5.915777845 | -1.121443628 | 1.121443628 |
|  | WWC2 | 5.092845419 | 7.079411902 | -0.993283241 | 0.993283241 |
|  | CCN2 | 4.950209213 | 5.981737168 | -0.515763977 | 0.515763977 |
|  | NFATC1 | 2.322688784 | 4.8575263 | -1.267418758 | 1.267418758 |
|  | SAV1 | 5.090617275 | 5.834013866 | -0.371698296 | 0.371698296 |
|  | LATS2 | 4.73598117 | 5.183238593 | -0.223628712 | 0.223628712 |
|  | AMOT | 8.03581852 | 5.694120324 | 1.170849098 | -1.170849098 |
|  | TEAD3 | 6.501054647 | 5.06108691 | 0.719983868 | -0.719983868 |
| ECM Organization | COL1A1 | 6.796472706 | 11.17617651 | -2.1898519 | 2.1898519 |
|  | COL1A2 | 7.036301216 | 10.17879705 | -1.571247915 | 1.571247915 |
|  | COL3A1 | 2.829705105 | 11.56771755 | -4.369006221 | 4.369006221 |
|  | FN1 | 9.438208711 | 12.09385987 | -1.327825581 | 1.327825581 |
|  | MMP2 | 4.166598254 | 7.540254754 | -1.68682825 | 1.68682825 |
|  | TIMP1 | 4.382334301 | 6.290153212 | -0.953909455 | 0.953909455 |
|  | BGN | 2.962354624 | 4.912675611 | -0.975160493 | 0.975160493 |
|  | FMOD | 0.144480489 | 5.525490159 | -2.690504835 | 2.690504835 |
|  | LOX | 0.144480489 | 5.525490159 | -2.690504835 | 2.690504835 |
| Cell-cell Adhesion | CDH11 | 3.635589811 | 6.917326464 | -1.640868326 | 1.640868326 |
|  | CLDN11 | 0.610381414 | 2.838035724 | -1.113827155 | 1.113827155 |
|  | GJA1 | 9.145070645 | 10.62511127 | -0.74002031 | 0.74002031 |
|  | CD9 | 6.350133056 | 7.488254471 | -0.569060708 | 0.569060708 |
|  | AMIGO2 | 1.706928289 | 4.773949249 | -1.53351048 | 1.53351048 |
|  | PCDH10 | 2.981721576 | 6.093359105 | -1.555818764 | 1.555818764 |
|  | DSP | 10.14728416 | 9.295705284 | 0.425789437 | -0.425789437 |
|  | CDH1 | 8.953460247 | 8.302594568 | 0.325432839 | -0.325432839 |
|  | JAM3 | 6.138872189 | 6.564341437 | -0.212734624 | 0.212734624 |
| Apical Junction and Epithelial Polarity | PODXL | 5.621826526 | 10.61088091 | -2.494527191 | 2.494527191 |
|  | ANXA8 | 0.869449691 | 4.724091443 | -1.927320876 | 1.927320876 |
|  | ANXA8L1 | 1.188801285 | 4.727417489 | -1.769308102 | 1.769308102 |
|  | VIL1 | 1.074499829 | 4.005610807 | -1.465555489 | 1.465555489 |
|  | CDHR1 | 2.162201745 | 4.134920669 | -0.986359462 | 0.986359462 |
|  | MYO1E | 5.024457091 | 6.541195076 | -0.758368992 | 0.758368992 |
|  | MUC1 | 4.232752963 | 2.414267486 | 0.909242738 | -0.909242738 |
|  | CLDN4 | 6.020579545 | 2.00476115 | 2.007909198 | -2.007909198 |
|  | MUC3A | 4.514532907 | 6.061176718 | -0.773321906 | 0.773321906 |
| Developmental Signaling Pathways | FZD7 | 5.385575944 | 6.997621975 | -0.806023015 | 0.806023015 |
|  | AXIN2 | 5.092845419 | 5.50967821 | -0.208416395 | 0.208416395 |
|  | FRZB | 3.367640988 | 7.932622466 | -2.282490739 | 2.282490739 |
|  | DKK1 | 0.839954875 | 4.525359969 | -1.842702547 | 1.842702547 |
|  | TGFB1 | 3.724494889 | 5.566190362 | -0.920847737 | 0.920847737 |
|  | SMAD7 | 3.487648118 | 4.624126445 | -0.568239163 | 0.568239163 |
|  | SULF1 | 0 | 4.271125387 | -2.135562693 | 2.135562693 |
|  | NLRP2 | 0 | 2.489940232 | -1.244970116 | 1.244970116 |
|  | PTCH1 | 0 | 2.109447318 | -1.054723659 | 1.054723659 |
|  | GLI3 | 7.401375242 | 5.655121271 | 0.873126986 | -0.873126986 |

Table S3: Representative inner ear development-associated genes identified by RNA sequencing and used for transcriptomic analyses.

| **Category** | **Gene Symbol** | **Normalized expression** | | **Log2 Fold change** | |
| --- | --- | --- | --- | --- | --- |
|  |  | U-hIEO | M-hIEO | U-hIEO | M-hIEO |
| Otic epithelium | EPHA7 | 5.179 | 6.587 | -0.703930254 | 0.703930254 |
|  | JAG1 | 7.261 | 5.855 | 0.70340184 | -0.70340184 |
|  | PKDCC | 2.851 | 4.951 | -1.050155279 | 1.050155279 |
|  | PAX2 | 0.000 | 0.593 | -0.29635923 | 0.29635923 |
|  | SPP1 | 4.088 | 6.035 | -0.973411074 | 0.973411074 |
|  | EMX2 | 5.625 | 0.957 | 2.333907157 | -2.333907157 |
|  | TBX1 | 2.116 | 3.333 | -0.608029212 | 0.608029212 |
|  | COL21A1 | 3.382 | 5.710 | -1.163741332 | 1.163741332 |
|  | COL14A1 | 2.439 | 6.238 | -1.899481554 | 1.899481554 |
|  | AKAP12 | 6.145 | 8.116 | -0.985232926 | 0.985232926 |
|  | KCTD12 | 5.397 | 7.203 | -0.903151592 | 0.903151592 |
|  | SLCO1A2 | 1.397 | 4.338 | -1.470963307 | 1.470963307 |
|  | CNMD | 0.507 | 6.010 | -2.751434871 | 2.751434871 |
|  | LMX1A | 0.000 | 1.142 | -0.570946447 | 0.570946447 |
| Nuerosensory | FST | 3.642 | 6.307 | -1.332857117 | 1.332857117 |
|  | EFNB2 | 7.896 | 6.347 | 0.774445694 | -0.774445694 |
|  | EGR1 | 3.701 | 6.129 | -1.213704492 | 1.213704492 |
|  | LDB2 | 2.338 | 6.052 | -1.856927846 | 1.856927846 |
|  | DSEL | 4.284 | 5.797 | -0.756341596 | 0.756341596 |
|  | TTYH1 | 4.666 | 3.465 | 0.600501217 | -0.600501217 |
|  | NFIA | 4.390 | 2.590 | 0.899825785 | -0.899825785 |
|  | APOE | 6.066 | 8.639 | -1.286649653 | 1.286649653 |
|  | SOX10 | 1.695 | 3.009 | -0.656943371 | 0.656943371 |
| Supporting cells | HES1 | 6.161 | 5.045 | 0.558309161 | -0.558309161 |
|  | SPARC | 9.031 | 10.826 | -0.897290107 | 0.897290107 |
|  | EMP3 | 2.411 | 4.481 | -1.035196574 | 1.035196574 |
|  | SMOC2 | 1.425 | 4.494 | -1.534233742 | 1.534233742 |
|  | TIMP1 | 4.382 | 6.290 | -0.953909455 | 0.953909455 |
|  | VAT1L | 3.953 | 5.789 | -0.918119817 | 0.918119817 |
|  | VASN | 2.797 | 4.305 | -0.753804081 | 0.753804081 |
|  | S100A13 | 4.589 | 5.596 | -0.50370654 | 0.50370654 |
|  | FOS | 3.110 | 4.744 | -0.816693698 | 0.816693698 |
|  | MYL9 | 4.636 | 6.249 | -0.80672558 | 0.80672558 |
|  | TRDN | 2.229 | 6.902 | -2.336477711 | 2.336477711 |
|  | RAB31 | 3.418 | 5.948 | -1.265065624 | 1.265065624 |
|  | FGFR3 | 0.000 | 1.500 | -0.750092829 | 0.750092829 |
| Hair-cell maturation and function | MYO7A | 0.000 | 2.378 | -1.188959991 | 1.188959991 |
|  | MYO6 | 1.925147863 | 4.486428648 | -1.280640392 | 1.280640392 |
|  | ATOH1 | 0.073194441 | 2.061961374 | -0.994383467 | 0.994383467 |
|  | ESPN | 2.850 | 3.613 | -0.38142974 | 0.38142974 |
|  | CALB1 | 1.611 | 4.660 | -1.524845062 | 1.524845062 |
|  | ATP1B1 | 5.910 | 7.116 | -0.603034886 | 0.603034886 |
|  | MYO1E | 5.024 | 6.541 | -0.758368992 | 0.758368992 |
|  | DIAPH2 | 4.342 | 5.678 | -0.668300659 | 0.668300659 |
|  | HK1 | 6.886 | 8.140 | -0.627276629 | 0.627276629 |
|  | TMC1 | 0.073194441 | 0.397861645 | -0.162333602 | 0.162333602 |
|  | KCNQ4 | 0.000 | 1.729 | -0.864275811 | 0.864275811 |
| Synaptic maturation | OTOF | 0.941555981 | 0.326586672 | 0.307484655 | -0.307484655 |
|  | STRIP2 | 1.951671594 | 2.868776628 | -0.458552517 | 0.458552517 |
|  | PCDH15 | 0.000 | 1.820 | -0.910109234 | 0.910109234 |
|  | CDH23 | 0.000 | 1.388 | -0.694022267 | 0.694022267 |
|  | EPHA7 | 5.179 | 6.587 | -0.703930254 | 0.703930254 |
